# Spontaneous DNA Synapsis by Forming Noncanonical Intermolecular Structures

**DOI:** 10.3390/polym14102118

**Published:** 2022-05-23

**Authors:** Viacheslav Severov, Vladimir Tsvetkov, Nikolay Barinov, Vladislav Babenko, Dmitry Klinov, Galina Pozmogova

**Affiliations:** 1 Federal Research and Clinical Center of Physical-Chemical Medicine, Malaya Pirogovskaya Str. 1a, 119435 Moscow, Russia; nik_mipt@mail.ru (N.B.); daniorerio34@gmail.com (V.B.); klinov.dmitry@mail.ru (D.K.); pozmge@gmail.com (G.P.); 2 Institute of Biodesign and Complex System Modeling, I.M. Sechenov First Moscow State Medical University, Trubetskaya Str. 8-2, 119991 Moscow, Russia; 3 A.V. Topchiev Institute of Petrochemical Synthesis, Leninsky prospect Str. 29, 119991 Moscow, Russia; 4 Peoples’ Friendship University of Russia (RUDN University), 6 Miklukho-Maklaya str.6, 117198 Moscow, Russia

**Keywords:** DNA-DNA junction, G-quadruplex, i-motif, recombination

## Abstract

We report the spontaneous formation of DNA-DNA junctions in solution in the absence of proteins visualised using atomic force microscopy. The synapsis position fits with potential G-quadruplex (G4) sites. In contrast to the Holliday structure, these conjugates have an affinity for G4 antibodies. Molecular modelling was used to elucidate the possible G4/IM-synaptic complex structures. Our results indicate a new role of the intermolecular noncanonical structures in chromatin architecture and genomic rearrangement.

## 1. Introduction

G-quadruplexes (G4s), noncanonical helical-nucleic acid structures composed of stacked G-tetrads are planar arrangements of four guanine residues bound through Hoogsteen base pairing and stabilised by cations, particularly potassium [1]. They adopt several topologies depending on DNA-strand orientation (parallel, antiparallel, or hybrid), G-tetrad number, and the number of involved strands (intra- vs. intermolecular). Wherever there is a G4-forming sequence in one DNA strand, the complimentary strand always contains [2] a C-rich sequence that can form another tetraplex structure. This is known as an i-motif (IM) and is composed of two parallel-stranded duplexes that are held together in an antiparallel orientation by intercalated C:CH^+^ base pairs.

Interest in G4s emerges from the growing evidence that they play several important roles in biology. Bioinformatic analyses have shown that putative G4 sites (PQSs) are not randomly distributed along the genome, but cluster at defined regions such as telomeric repeats [3], immunoglobulin switch regions [4], replication origins [5], recombination sites [6,7], gene promoters [8], and copy number variation breakpoints [9]. PQSs are also often present in specific parts of transposable elements [10], particularly in human Alu repeats [11,12]. More than 700,000 PQSs have been found in the human genome [13]. This number may be significantly broadened to include imperfect G4s [14] or G4s consisting of guanines from both strands of genomic DNA [15]. They are involved in processes such as transcription [16,17,18], replication [19], recombination [7,20], and genome instability [21,22,23]. The formation of G4 conformations in the human genome has been visualised in vivo [24,25].

IMs have long been considered a structural curiosity that cannot exist under physiological conditions (IMs are typically stable only at mildly acidic pH due to the requirement for cytosine hemiprotonation, while pH in the nucleus equals ~7.3). However, recent findings indicate that conditions such as negative superhelicity or molecular crowding facilitate IM formation [2]. Moreover, sequences with at least five cytosine tracts (thousands of cytosine-rich sequences are present in the human genome) may fold into IM at physiological pH even in the absence of superhelicity and crowding [26]. Finally, in vivo IM formation has been visualised in the human nucleus using IM-specific antibodies [27].

Research on biologically relevant G4s has mainly focused on monomolecular structures usually using ssDNAs. Although the G4 and IM structures are well characterised, little is known about their behaviour in dsDNA. Intermolecular noncanonical DNA structures are gaining increasing attention. However, studies in this field are usually performed on model oligonucleotides [28,29], and investigations of native intermolecular G4s are limited to RNA:DNA hybrids that are formed during transcription [30]. Many G4-related processes, such as recombination, enhancer-promoter interactions [31], chromatin remodelling [32], and chromosomal rearrangements require a close approach or even physical contact of two DNA chains (or two remote parts of one DNA chain), that is, DNA-DNA synapsis/junction. The participation of various protein factors in these processes is described in detail with this passive part being assigned to DNA strands. Simultaneously, the polynucleotide nature allows DNA to form different conformational structures depending on the conditions.

The idea that the formation of intermolecular G4s may provide DNA-DNA contacts in vivo was first proposed in [33]. The authors showed self-association of ssDNA containing G-rich motifs under physiological salt concentrations. In a series of articles [34,35,36], the formation of synaptic complexes by artificial synapsable duplexes with non-complementary “sticky” G-domains was investigated. However, it remains unclear whether the synaptic complexes can be formed by native duplexes composed of fully complementary strands, one of which is G-rich and capable of intermolecular G4 formation. Moreover, it is unknown whether synaptic contacts can be formed by the same duplexes via C-rich strands due to the formation of intermolecular IMs. Obviously, the rearrangement of native dsDNAs into such structures is not straightforward, so it is difficult to reveal them using methods such as NMR or optical methods. Atomic force microscopy (AFM) is a technique for the direct visualisation of single biological molecules. This method has been used previously to visualise DNA duplexes and triplexes [37], G4s and G-wires [28,38,39], IMs [29], and synapsable quadruplex-mediated fibres [40].

In this work, we used AFM to visualise natural dsDNA fragments containing well-known G4s from the human genome (*cMyc* [41] and *KRAS* [42] promoter regions) and the genome of *N. gonorrhoeae* (pilin expression locus [7]), as well as model (designed in-house) 195 bp DNA duplexes containing (G_3_T)_n_G_3_ sequences (n = 1–5) in the middle regions. AFM scanning of the duplexes revealed intermolecular cruciform and higher-order structure formation that allowed us to assume G4/IM-synaptic complex formation. No signs of such complexes were visible in the AFM images of the control duplexes that lack PQS or its part. The presence of G4 folding in the core of the formed complexes was confirmed by an anti-G4-DNA antibody (clone 1H6) [24,25]. Possible nucleotide folding in G4s and IMs, the geometry of G4 and IM arrangement relative to each other, as well as the stability of the formed synaptic complexes were analysed using molecular modelling techniques. Based on the AFM results we also suggest a mechanism of synaptic complex-promoted DNA strand exchange (recombination).

## 2. Materials and Methods

### 2.1. Synthesis, Purification, and MS Characterisation of Oligonucleotides

Oligonucleotides (ONs) (Table 1) were synthesised using a Biosset ASM-800 DNA synthesiser (Biosset Ltd.; Novosibirsk, Russia) and standard reagents (Glen Research;Sterling, VA, USA), following standard phosphoramidite protocols. For synthesising 5′-phosphorylated ONs, solid CPR II (Glen Research) was used. 5′-dimethoxytritylated (DMT) ONs were purified using preparative-scale reverse-phase high-performance liquid chromatography (HPLC) on a 250 × 4.6 mm Hypersil C18 column (Thermo Fisher Scientific; Waltham, MA, USA) with detection at λ = 260 nm and a linear 7.5–25% acetonitrile gradient in 0.1 M ammonium acetate buffer over 45 min at 50 °C, flow rate: 0.85 mL/min. DMT-protection groups were removed by treatment with 80% acetic acid for 30 min and 5′-phosphorylated ONs after detritylation were treated with 32% ammonium hydroxide for 15 min to eliminate the side chains from 5′-phosphate, according to the manufacturer’s instructions. The detritylated ONs were further HPLC-purified in 4–11.5% acetonitrile gradient in 0.1 M ammonium acetate buffer, ethanol precipitated, and dissolved in 1 × TE buffer (10 mM Tris, 1 mM EDTA; pH 8.0) to reach a final concentration of 10 mM. The purity of all ONs was determined to be ≥ 95% using HPLC. Matrix-assisted laser desorption ionisation time-of-flight (MALDI TOF) mass spectrometry was used to verify the compliance of theoretical and experimental ON masses, as described previously [28]. The observed difference between the theoretical and experimental ON masses was less than 3 Da (Table 1).

### 2.2. Amplification of Human and N. Gonorrhoeae DNA Fragments Containing PQS

For amplification of cMyc (201 bp), kRas (208 bp), and control 0Myc (200 bp) duplexes, human total genomic DNA [43] was used as template, and *N. gonorrhoeae* (FA1090 strain) total genomic DNA [43] was used as a template for NG duplex (200 bp) production. Amplicons cMyc and NG were amplified using Taq polymerase (Lytech; Moscow, Russia), and kRas was amplified using the Encyclo GC polymerase kit (Evrogen; Moscow, Russia), and control duplex 0Myc with a Screen Mix-HS polymerase kit (Evrogen). Amplifications were performed using a S1000TM thermal cycler (Bio-Rad; Hercules, CA, USA) under the following conditions: initial denaturation at 97 °C for 3 min, followed by 35 cycles of denaturation at 97 °C for 15 s, annealing at respective temperatures for each primer set (61 °C for R_NG/F_NG and R_kRas/F_kRas, 65 °C for R_cMyc/F_cMyc, and 59 °C for R_0Myc/F_0Myc primer pairs) for 10 s, and elongation at 72 °C for 15 s. The PCR products were separated using electrophoresis on a 2% agarose gel. The amplicons of proper size were excised, gel-purified using the Cleanup Standard kit (Evrogen), according to the manufacturer’s instructions and washed from the membrane with buffer containing 10 mM Tris-HCl, pH 5.6, and 10 mM KCl (AFM buffer).

### 2.3. Construction of Model DNA Duplexes for AFM

DNA duplexes 0, 2m, 3m, 4m, 5m, and 6m (Table S1) were constructed through a two-step PCR using a S1000TM thermal cycler (Bio-Rad). The first PCR amplification was performed in 20 µL reaction mixture, containing 2 µL Lig1, Lig2, Pr, Pf, 5′-fl and 3′-fl, 2 µL 10^x^-dNTPs (Lytech), 2 µL 10^x^-Pfu buffer (100 mM Tris-HCl, pH 8.85, 250 mM KCl, 50 mM (NH_4_)_2_SO_4_, 20 mM MgSO_4_, 1% Tween 20; (α-Ferment; Moscow, Russia) or hand-made buffer containing 200 mM Tris-HCl, pH 8.6; 200 mM LiCl, 25 mM MgCl_2_, 2 µL middle region DNA part (mid_0, mid_2, mid_3, mid_4, mid_5, or mid_6, respectively), 1.5 µL nuclease free water, and 0.5 µL (5 U/µL) Pfu TURBO polymerase (α-Ferment). The reactions were subjected to 20 amplification cycles using the following cycling programme: 94 °C for 10 s, 37 °C for 10 s, and 68 °C for 30 s. The second PCR amplification was performed in 25 µL reaction mixture containing 2.5 µL 10^x^-Pfu buffer or hand-made Li^+^-based buffer, 2.5 µL 10^x^-dNTPs, 0.5 µL first PCR mixture, 1 µL primers Pr and Pf, 17 µL nuclease free water, and 0.5 µL Pfu TURBO polymerase. The reactions were subjected to 30 amplification cycles, according to the following cycling programme: 94 °C for 10 s, 50 °C for 15 s, and 68 °C for 30 s. The PCR products were analysed using 10% denaturing (7 M urea) PAGE. The gels were stained with SYBR Gold (Thermo Fisher Scientific) and analysed using a Gel Doc scanner (Bio-Rad). The PCR products were separated using electrophoresis on a 2% agarose gel. The amplicons of proper size were excised, gel-purified using the Cleanup Standard kit (Evrogen), according to the manufacturer’s instructions and washed from the membrane with AFM buffer (10 mM Tris-HCl, pH 5.6; and 10 mM KCl).

### 2.4. Non-Denaturing PAGE

DNA duplexes solutions (≈1 mM) in AFM buffer were stored at 4 °C overnight, then loaded on 10% non-denatured PAGE (5µL per well). The gels were run for 2 h at 200 V, stained with SYBR Gold and analysed using a Gel Doc scanner.

### 2.5. Sanger Sequencing

The amplicon sequences were obtained through the Sanger dideoxy sequencing method using a Big DyeTM Terminator v.3.1 Cycle Sequencing Kit and ABI Genetic Analyzer 3500XL, according to the manufacturer’s instructions (Applied Biosystems; Waltham, MA, USA).

### 2.6. The Design of an Asymmetric Holliday Junction

Oligonucleotides Hol-1, Hol-2, Hol-3, and Hol-4 were slowly annealed from 97 °C to 45 °C in 80 µL buffer containing 20 mM KCl and 10 mM Tris-HCl, pH 7.6 (1.25 pmol/µL each DNA chain). Hol-fl and Hol-fs (5 pmol/µL each DNA chain) solution in 80 µL of the same buffer was also annealed to 45 °C. Subsequently, these two solutions were quickly mixed and slowly annealed to room temperature (≈25 °C). The folded structure was ligated using T4 DNA ligase (Thermo Fisher Scientific), according to the manufacturer’s instructions. The Holliday junction was separated analogically to the PCR products and dissolved in AFM buffer or AFM buffer supplemented with 2 mM MgCl_2_.

### 2.7. AFM Sample Preparation, Image Acquisition and Processing

AFM were performed on freshly cleaved graphite surfaces rendered hydrophilic with an amphiphilic modificator (CH_2_)_n_(NCH_2_CO)_m_-NH_2_ [37]. DNA samples were diluted 20–40 times with AFM buffer, applied on the substrate surface, incubated for 5–15 s, and removed with a nitrogen stream, thus drying the surface for imaging in air. The low salt concentration of the dilution buffer allowed us to eliminate a rinsing step from the sample preparation procedure which could otherwise alter the folding of synaptic structures. All experiments were performed at least in triplicate. AFM imaging was performed using a multimode AFM instrument with an NTEGRA Prima controller (NT-MDT; Russia) in tapping mode with a 1 Hz scan rate and a typical free amplitude of several nanometres. All measurements were performed in air using supersharp cantilevers grown on the tips of commercially available standard silicon cantilevers using a chemical vapour deposition process (spike diameter: approximately 1 nm) [37]. FemtoScan Online software (ATC; Moscow, Russia; http://www.femtoscanonline.com, accessed on 17 August 2012) was used to filter and present the AFM data. Standard algorithms for AFM image flattening were used (subtracting the quadric surface and averaging by lines), and no algorithms for resolution improvement were used. Thus, the raw AFM images are presented in this study. Image Magic software (SPM; London, UK; https://sites.google.com/site/spmimagemagic, accessed on 5 May 2022) was used to semi-automatically analyse the ON heights. The analysis consisted of two steps: the individual particles were identified automatically on the images by the local maxima and their heights were calculated with respect to the local background surrounding the particles. The results of the automatic analysis were filtered manually when necessary.

### 2.8. Molecular Modelling and Molecular Dynamic Simulation

All 3D models of the studied structures were built using the molecular graphics software package Sybyl-X software (Certara; USA) using the following strategy. Initially, models of the required duplexes, quadruplexes, and IMs were created. Further, the created models were located relative to each other in the required geometry and connected. At each stage, molecular mechanical optimisation was performed to eliminate the van der Waals overlap which could occur during a certain step. The molecular mechanical optimisations were performed using Sybyl-X and Powell’s method with the following settings: the parameters for intermolecular interactions and the values of partial charges were taken from force field amber7ff99 with a non-bonded cut-off distance of 8 Ǻ. The effect of the medium was a dielectric constant of 4 and the number of iterations was 1000, according to the simplex method for initial optimisation and a 0.05 kcal*mol^−1^*Å^−1^ energy gradient convergence criterion. The stability of the created models was tested by molecular dynamics using the Amber 20 software [44]. The MD simulations in the production phase were performed using constant temperature (T = 300 K) and pressure (*p* = 1 atm) over 50 ns. To control the temperature, a Langevin thermostat was used with 1 ps^−1^ collision frequency. The influence of the solvent was simulated with the application model of water molecules OPC3 [45]. K^+^ ions were used to neutralise the negative charge of the DNA backbone. The parameters needed for the interatomic energy calculation were taken from the force fififields OL15 [46,47].

The free energy was calculated as the sum of the electrostatic energies (E_q_), Van der Waals energies (E_VDW_), the energy of solvation and the deformation energy of valence bonds, and the valence and dihedral angles (U). The energy of solvation was calculated as the sum of the polar and nonpolar contributions. The polar contribution (E_GB_) was computed using the Generalized Born (GB) method and the algorithm developed by Onufriev et al. for calculating the effective Born radii [48]. The non-polar contribution to the solvation energy (E_surf_) which includes solute-solvent van der Waals interactions and the free energy of cavity formation in solvent was estimated from a solvent-accessible surface area (SASA).

## 3. Results

### 3.1. G4/IM-Synaptic Structure Formation by DNA Duplexes Containing PQS

To verify the possibility of synaptic complex formation between native DNA duplexes we studied DNA duplex fragments of human and *N. gonorrhoeae* genomes (≈200 bp) with PQS in its middle regions using a high-resolution AFM. DNA samples containing well-known G4s of two oncogene promoters were chosen. These were as follows: cMyc duplex (201 bp), including Pu27 PQS of cMyc promoter NHE III_1_ element [41]; kRas duplex (208 bp), including PQS in GA-element of KRAS gene promoter [42]; and G4-forming sequence located upstream of the *N. gonorrhoeae* pilin expression locus (NG duplex, 200 bp) required for pilin antigenic variation [7]. A 0Myc (200 bp) sequence that was located near the cMyc fragment of the human genome and without PQS was used as a control. The sequences are listed in Table S1.

The DNA samples were obtained through PCR amplification of the human or N. gonorrhoeae (FA1090 strain) total genomic DNA [43]. The primers (Table 1) were selected using the Primer-BLAST tool (https://www.ncbi.nlm.nih.gov/tools/primer-blast/, accessed on 18 November 2011). The amplicons were separated by agarose gel electrophoresis and dissolved in a buffer containing 10 mM Tris-HCl (pH 5.6) and 10 mM KCl (AFM buffer).

**Figure 1:**
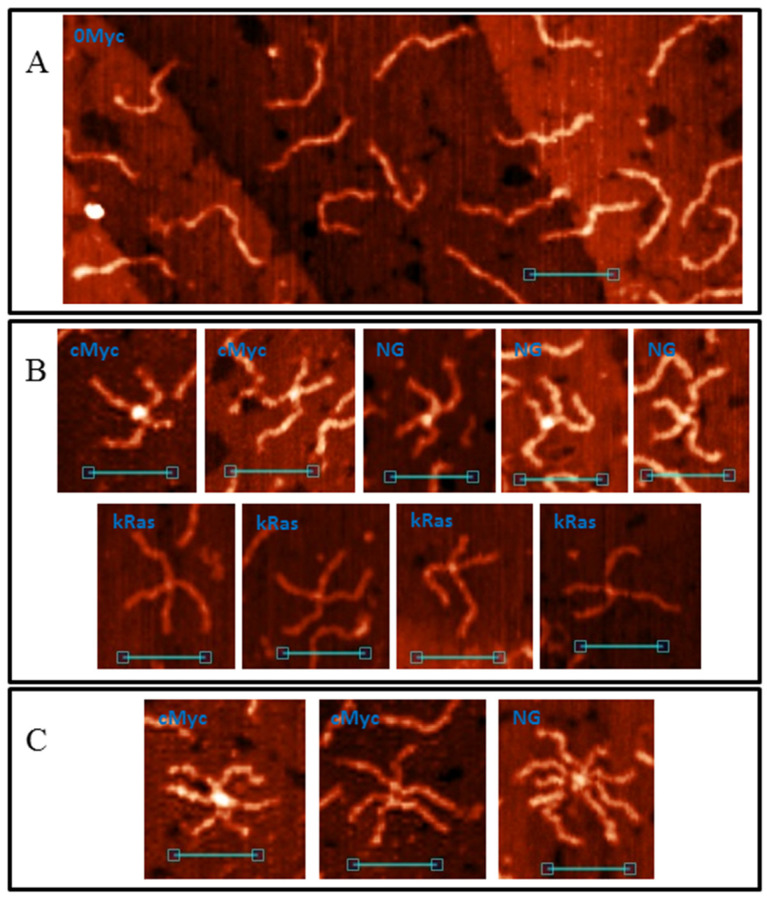
AFM images of natural dsDNA fragments. (**A**) AFM image of 0Myc duplex; (**B**) Cruciform structures, formed by PQS-containing duplexes; (**C**) Complex G4/IM-synaptic complexes formed by more than two molecules. Scale bars: 50 nm.

An AFM image of the control duplex 0Myc is shown in Figure 1A. As seen in the figure, only separate DNA molecules (height 1.0 ± 0.1 nm and length 65 ± 3 nm) were found which corresponds with the DNA length in the solution [49]. Some of the molecules had melted areas, such as single-stranded loops and tails. This is the main difference between the molecules.

DNA samples cMyc, kRas and NG, containing PQSs in their middle regions are also presented mostly by separate molecules, but with this the images contained cruciform (Figure 1B) and higher-order structures (Figure 1C). We called them the G4/IM-synaptic complexes. The measured heights of the central cores varied from molecule to molecule and showed elevation in comparison to the duplex arms from complete absence of to 2.5 nm. Thus, the two DNA duplexes may form different structure junctions. In addition, some complexes are not joined by their middle regions, which are the PQSs. These sequences are GC-rich and contain areas where two or more G3-tracks are divided by less than seven nucleotides. They may also form synaptic complexes through intermolecular G4 or IM.

It is reasonable to assume that increasing the number of G_3_-trackts leads to increasing the number of possible types of synaptic complex structures. To verify this suggestion we synthesised a model set of 195 bp DNA duplexes containing varying numbers of G_3_-tracks ((G_3_T)_n_G_3_, n = 1–5) within statistical duplex media. The (G_3_T)_n_G_3_ sites are located in the middle of the duplexes, so the formed synaptic complexes must be symmetrical (have duplex arms of equal lengths). This set of samples with varying numbers of G_3_-trackts is necessary to show the increase in the complexity and variety of the possible structures from the simplest (formed by 2m) to the most difficult (6m) and to correlate the results of AFM with molecular models.

### 3.2. Synthesis of DNA Constructs for AFM

**Figure 2:**
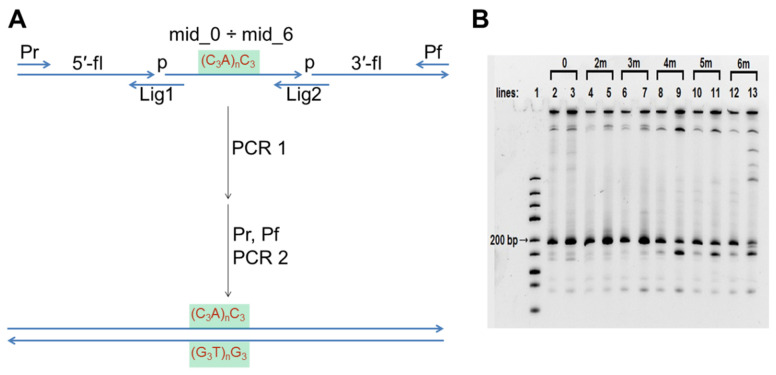
Synthesis of model DNA duplexes 0, 2m, 3m, 4m, 5m, and 6m. (**A**) Scheme of two-step PCR synthesis of model DNA constructs; (**B**) Analytical PAGE of amplification. Line 1–DNA ladder mix, the nearest to desired 195 bp length band (200 bp) is marked. Lines 3, 5, 7, 9, 11 and 13–PCR in the commercial buffer; lines 2, 4, 6, 8, 10 and 12–PCR in the in-house Li-based buffer.

The sequences of 0, 2m, 3m, 4m, 5m, and 6m dsDNA-constructs are given in Table S1. They differ only in their middle section, so they were obtained using a universal two-step PCR (Figure 2A, oligonucleotides used for amplification are shown in Table 1). The analytical polyacrylamide gel electrophoresis (PAGE) of this two-step synthesis is shown in Figure 2B. Lines 3, 5, 7, 9, 11, and 13 show the results of the PCR that was conducted using a commercial buffer (100 mM Tris-HCl, pH 8.85, 250 mM KCl, 50 mM (NH_4_)_2_SO_4_, 20 mM MgSO_4_, and 1% Tween 20). A PCR amplification of 0, 2m, and 3m was efficient under such conditions, but the efficiency decreased significantly when amplicons contained sequences that were capable of intramolecular G4 folding (4m, 5m, and particularly 6m). Moreover, we observed a discrete ~100 bp product formation along with the expected 195 bp amplicon (lines 9, 11, 13). A similar appearance of short PCR products was observed during amplifying the *vlsE* region from *B. burgdorferi* [20], and it is common knowledge that the sequences containing PQS are difficult PCR templates. Amplification of such sequences (particularly construction of PQS-containing duplexes from oligonucleotides using PCR) often requires intensive optimisation and/or the use of PCR additives [50]. The efficiency of the synthesis turned out to be insufficient for the production of 4m, 5m, and particularly 6m samples for AFM investigation.

PCR buffer conditions (pH > 8) generally prevent IM formation, but the PCR buffer usually contains K^+^ and NH_4_^+^ salts which facilitate G4 folding. G4s are reportedly unstable in Li^+^ salts [51]. Therefore, we substituted all K^+^ and NH_4_^+^ salts with LiCl and performed the PCR amplification in a buffer with 20 mM Tris-HCl, pH 8.6, 20 mM LiCl and 2.5 mM MgCl_2_. Overall, the amplification efficiency for sequences without PQS in this buffer was lower than in the commercial buffer (compare lines 2 and 3, 4 and 5, 6 and 7). However, the difference between the yields of amplicons with PQS (4m, 5m, and 6m) and without it (0, 2m, and 3m) was much less pronounced in the Li-based buffer (compare lines 2, 4, 6, 8, 10, and 12). In other words, the yields of PQS-containing amplicons was improved. This finding may be used to address the problems associated with allele dropout during the PCR of single nucleotide polymorphisms containing PQS [50]. After amplification in the Li-based buffer, duplexes 0, 2m, 3m, 4m, 5m, and 6m were separated using agarose gel electrophoresis and dissolved in AFM buffer. Similarly, duplex 5s with PQS not in the middle, but shifted, was obtained: 5-fl_5, mid_0, and 3′-fl were used as the main building blocks in the first PCR step.

### 3.3. Structure of G4/IM-Synaptic Complexes

DNA duplex 0 without G3-tracts in its sequence was used as a control. Its AFM images are similar to those of 0Myc molecules. The control images indicate the presence of 62 ± 3 nm-long separate DNA molecules which may contain melted areas.

**Figure 3:**
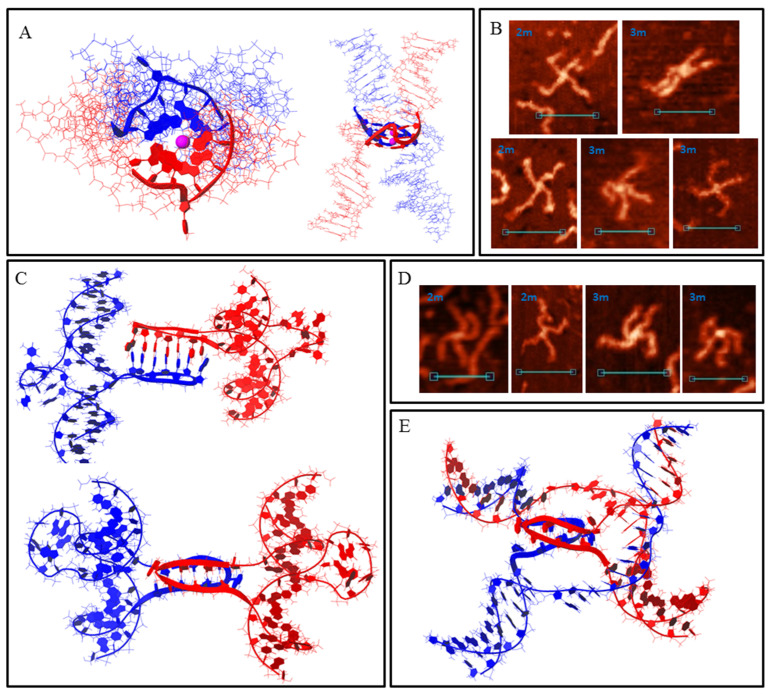
Bimolecular G4/IM-synaptic complexes formed by 2m and 3m samples. (**A**) Molecular model of duplex joining through intermolecular G4 folding. (**B**) Examples of the complexes consistent with model (**A**), revealed by AFM. Scale bars: 50 nm. (**C**) Molecular model of the joining through intermolecular IM folding. (**D**) Examples of the complexes consistent with model (**C**), revealed by AFM. Scale bars: 50 nm. (**E**) Molecular model of possible intermolecular IM formation upon Holliday structure moving.

DNA duplexes 2m and 3m may form synaptic complexes with two of the simplest structures, where the duplexes join through intermolecular G4 or IM formation. Molecular modelling of the first case (joining through G4) is shown in Figure 3A (hereafter, noncanonical shapes are highlighted with wider rendering. K^+^ ions which stabilise G4s are highlighted in magenta). Such structures must have a stiff G4 core and duplex arms must disperse symmetrically. Most of the synaptic complexes, visualised by AFM, are of this geometry (Figure 3B) with a 1.1–1.4 nm core structure height. Angles between the duplex arms vary and depend on the manner in which the molecular associate lies on the substrate surface. The morphology of a stable synaptic complex with joining through IM differs from that described above as the folded IM serves as a dividing bridge between two duplexes (Figure 3C). AFM images of these molecular associates are shown in Figure 3D. The core part dividing the duplexes was 5–7 nm long and had a 1.2 ± 0.1 nm height. Molecular modelling also revealed another possible stable IM structure in duplex media (Figure 3E). The formation of such a structure is impossible in this study because of the need for interlacing DNA chains, but theoretically it may fold while the Holliday structure is moving, thus influencing the recombination processes. The difference between 2m and 3m in the formation of such simple two-duplex complexes is that both available G_3_ (or C_3_) blocks in the 2m sample participate in synaptic structure formation; however, a different combination of two of the three blocks in the 3m sample may form the same synaptic structure. Moreover, the resolution of AFM is not sufficient to discriminate between them.

**Figure 4:**
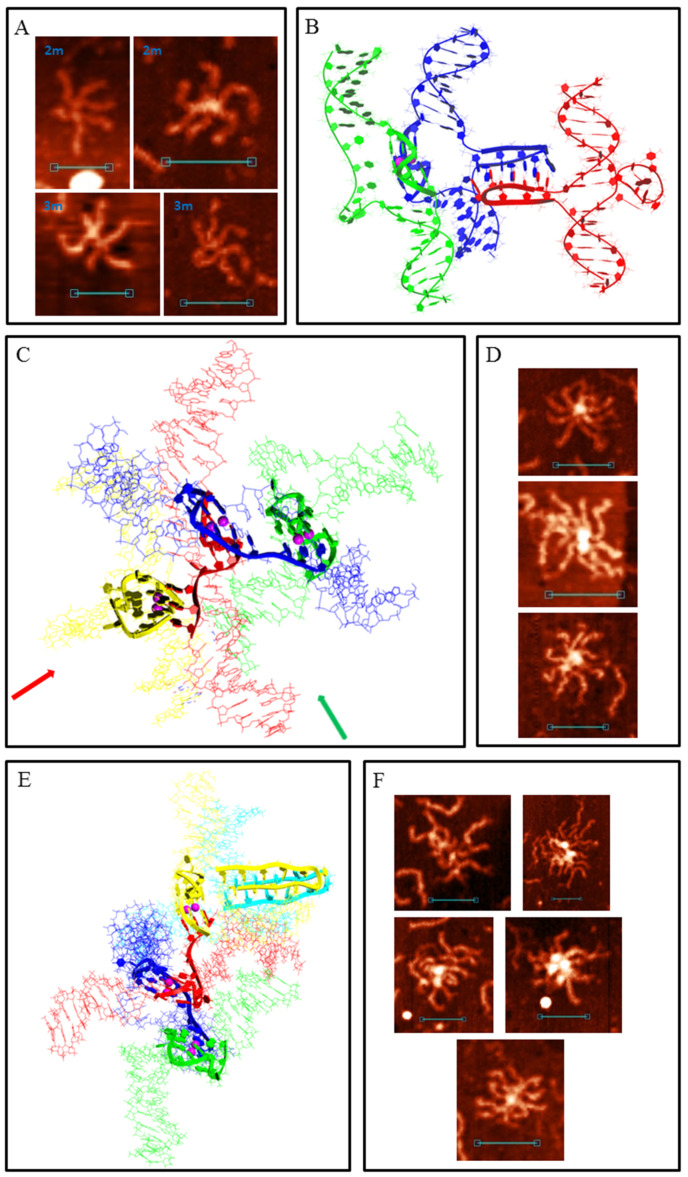
Multimeric G4/IM-synaptic complexes formed by 2m and 3m samples. (**A**) Examples of trimolecular complexes formed through intermolecular G4 and IM folding. Scale bars: 50 nm. (**B**) Molecular model of the structure, consistent with (**A**). (**C**) Molecular model of the tetrameric complex formed by 3m sample through the formation of three intermolecular G4s. The red and green arrows show the viewpoints from which the complexes are visible in the following examples. (**D**) Examples of the tetrameric complexes, revealed by AFM. Scale bars: 50 nm. (**E**) Molecular model of the synaptic complex formed by additional duplex joining with the previous structure through intermolecular IM formation. (**F**) Examples of the complexes, consistent with model (**E**), revealed by AFM. Bottom image represents the only example of the complex that remained intact (was not disrupted) during the AFM experiment. Scale bars: 50 nm.

When one G-rich (or C-rich) chain of a DNA duplex participates in a synaptic complex formation the other free C-rich (or G-rich) chain may interact with the third duplex molecule, thus forming a multimeric G4/IM-synaptic complex. Such structures that are formed of three duplexes were found in the 2m and 3m samples (Figure 4A). These associates had clearly distinguishable gaps within the duplex junctions. They were also not symmetrical as one core part was stiffer and higher, while the other was lengthy. A stable structure that fully corresponded to the AFM images is shown in Figure 4B. Molecular modelling explains the gap between the two folds and predicts that G4 is located perpendicular to the IM. Theoretically, the joining of the next molecules and the synaptic complex growth may go further by the described method (for example, the model of the tetramolecular junction is shown in Figure S1A), but we did not find such large complexes for the 2m sample. This may be related to the insufficient stability of such large complexes under AFM conditions. However, multimeric structures formed of more than three duplexes were revealed for the 3m sample. Moreover, they were formed more often even than the smaller complexes described above. Molecular modelling predicts another possibility of joining, where it forms a G4 chain with no need for IM folding. When the synaptic complex is composed of three duplexes, the synaptic core contains two G4s, the first of which consists of three G_3_ blocks of the first duplex molecule and one of the second, while the remaining two G_3_ blocks of the second duplex form G4 with the two G_3_ blocks of the third duplex (Figure S1B). The G4s in this case were in close proximity and perpendicular to each other. Trimolecular associates of such geometry were not revealed by AFM, but tetramolecular complexes folded according to the same logic of G4 chain formation (Figure 4C) were found (Figure 4D). The central G4 was formed by two G_3_ blocks from each central duplex. The remaining third blocks formed side G4s with three G_3_ blocks of side duplexes. Eight-arm synaptic complexes have 1–3 G4 cores depending on the way the molecular associate lies on the substrate surface. At the front view of the associate (as shown in Figure 4C), all three G4s were distinguishable and had a height of 1.4–1.6 nm (bottom panel in Figure 4D). The green arrow indicates the viewpoint from which the complex appears, as can be seen in the middle AFM scan with core heights of 1.6–2 nm. The red arrow indicates the viewpoint where the three G4s lie on each other (upper AFM scan, core height 2.8 nm). Further synaptic complex growth by this scheme is impossible, but there is no steric hindrance for the free C-rich chains of side duplexes to form IM with the next duplexes (Figure 4E), thus forming stable five- or even six-duplex complexes. Most of these large complexes were disrupted during absorption to the substrate surface or by cantilever while scanning, and they were observed as a shapeless mixture of synaptic complexes (Figure 4F). However, one good example confirming this possibility was found (Figure 4F, bottom panel).

**Figure 5:**
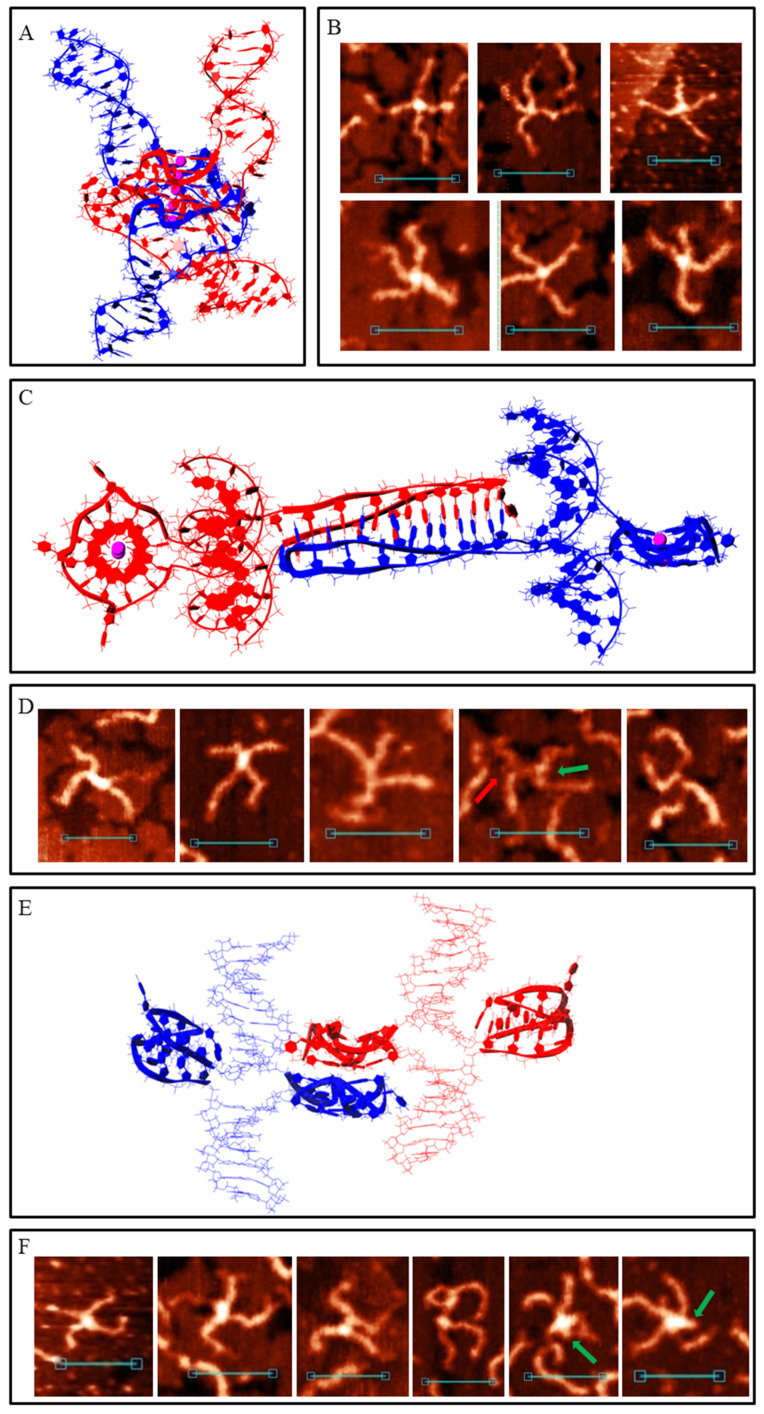
Bimolecular G4/IM-synaptic complexes formed by 4m sample. (**A**) Molecular model of duplex joining through interlocked G4-dimer folding. (**B**) Examples of the complexes, consistent with model (**A**), revealed by AFM. Scale bars: 50 nm. (**C**) Molecular model of duplex joining through two intermolecular IMs. (**D**) Examples of the complexes, consistent with model (**B**), revealed by AFM. Red arrow indicates the free G-rich chain, and green arrow indicates the folded G4 structure. Scale bars: 50 nm. (**E**) Molecular model of duplex joining through stacking of intramolecular G4s. (**F**) Examples of the complexes, consistent with model (**E**), revealed by AFM. Green arrows indicate folded IM structures. Scale bars: 50 nm.

Sample 4m may form all the synaptic complexes formed by 2m and 3m duplexes, and many such complex types (as bimolecular and multimeric) with the same parameters (core length and height) have been observed using AFM (Figure S2). With this, by the same scheme/logic of bimolecular synaptic complex formation via intermolecular G4 or IM folding, 4 m duplexes may join through more complex structures where all four G_3_ or C_3_ blocks participate. Molecular modelling of this association with the help of G-rich chains is shown in Figure 5A. Joining involves the formation of an interlocked G4-dimer whose structures at the single-chain level have previously been thoroughly described [28]. Residual single-stranded C-rich chains wrap the G4-core. In the AFM images (Figure 5B), such complexes are similar to those described above for joining through G4 (compare with Figure 3B). However, in this case the core was larger and had a 1.6 ± 0.2 nm height. Similarly, instead of intermolecular IM formed by 2m and 3m samples (Figure 3C), the presence of the fourth C_3_-block lead to folding of two IMs, divided by bulged thymidine residues that are between them. Molecular modelling (Figure 5C) predicts that thymidines do not fold into a tetrad or any other structured form. It also predicts that the released G-rich chains can fold into intramolecular G4s opposite intermolecular IMs. AFM images of synaptic complexes referring to this structure are shown in Figure 5D. The core part had a 10 ± 2 nm length and a 1.2 ± 0.1 nm height. It is unclear whether G4 folded in most scans, but in the fourth AFM image (from left to right) the molecular associate fell into a surface gap which corresponds to its core size. This makes border structures distinguishable from the central 2IMs part. It is clearly visible that one G-rich chain formed no secondary structure (red arrow) and the second formed a discernible globule (G4, green arrow). Therefore, we concluded that G4 formation may occur in this synaptic complex type. Another feature of the structure is the twist of the 2IM bridge which is not notable in AFM images because synaptic complexes tend to press down to the substrate surface during the AFM experiment. However, we found one clearly visible twist (fifth image).

The possibility of intramolecular G4 or IM formation expands the diversity of possible synaptic complex structures. This opens the way for another assembly type through the stacking of intramolecular G4s. For complexes of this type, molecular modelling predicts that the released C-rich chains can fold into IMs (Figure 5E). Previously, it has been shown that G4 and IM formation is mutually exclusive in duplex media [52]; however, this has not been studied in detail. In this work, the PQS sequence is another, and theoretically, their simultaneous formation is possible, at least in synaptic complex structures. The most stable stacking conformation occurs when the duplexes are the most distant, so they (and possible IMs) are opposite to each other, relative to the G4–G4 core. AFM images of such assemblies must be (in case of IMs that are not folded) similar to the previously described case of joining through intermolecular G4 (Figure 3B). We recognise them because G4 stacks are less stable than G4 structures and tend to disrupt at the substrate surface. Consequently, we observed different stages of stack decay (Figure 5F). The height of the G4 cores (irrespective of decay or stacking level) is 1.2–1.4 nm. When the complex was not disturbed (fifth and sixth AFM images), at least one of the two IMs folded (green arrows). The height of the central cores in this case was 1.4–1.6 nm, while that of the side IMs was ≈1.2 nm. It should be noted that no IM was observed opposite to the folded G4 in structures with disrupted stacking.

**Figure 6:**
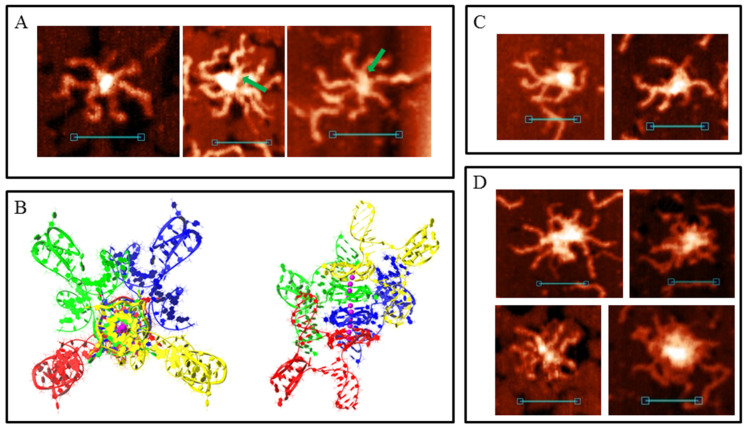
Multimeric synaptic complexes formed by 4m sample. (**A**) Examples of the complexes formed through stacking of four intramolecular G4s, revealed by AFM. Green arrows indicate folded top IM structures. Scale bars: 50 nm. (**B**) Molecular model of the structure, consistent with (**A**) (top and side views). (**C**) Tetramolecular synaptic complexes formed by 4m sample through a combination of G4-G4 stacking and the formation of two intermolecular IMs separated by thymidine residues. (**D**) Big complexes with indistinguishable structures. Scale bars: 50 nm.

The diversity of multimeric synaptic complex structures grows too in the case of a 4m duplex. Of those observed for the 2m and 3m samples, only the trimolecular associates formed by G4 and IM folding were found here (Figure S2). There were also centrally symmetric assemblies with 7–8 duplex arms and an 1.8–2.2 nm core height (Figure 6A). We ascribed them to the tetrameric complex formed completely due to intramolecular G4 stacking (Figure 6B). This structure explains the absence of the eighth arms in most cases as they lie down and are hidden from the cantilever during AFM. For the same reason, only the upper IM was visible (green arrow). Trimolecular assemblies formed through stacking were not revealed by AFM and molecular modelling confirmed that such associates are not stable. Therefore, we concluded that tetramolecular complexes are not built by the consecutive joining of duplexes but may form only by stacking two dimers. In this case, the central stacking occurs perpendicularly (the view from the top is symmetrical). Another peculiar tetrameric complex is shown in Figure 6C. We did not make a model of its structure, but it is clearly visible that it was folded by a combination of G4–G4 stacking and 2IMs (as in Figure 5C). Therefore, different methods of joining lead to the formation of large multimeric complexes with indistinguishable structures (Figure 6D).

**Figure 7:**
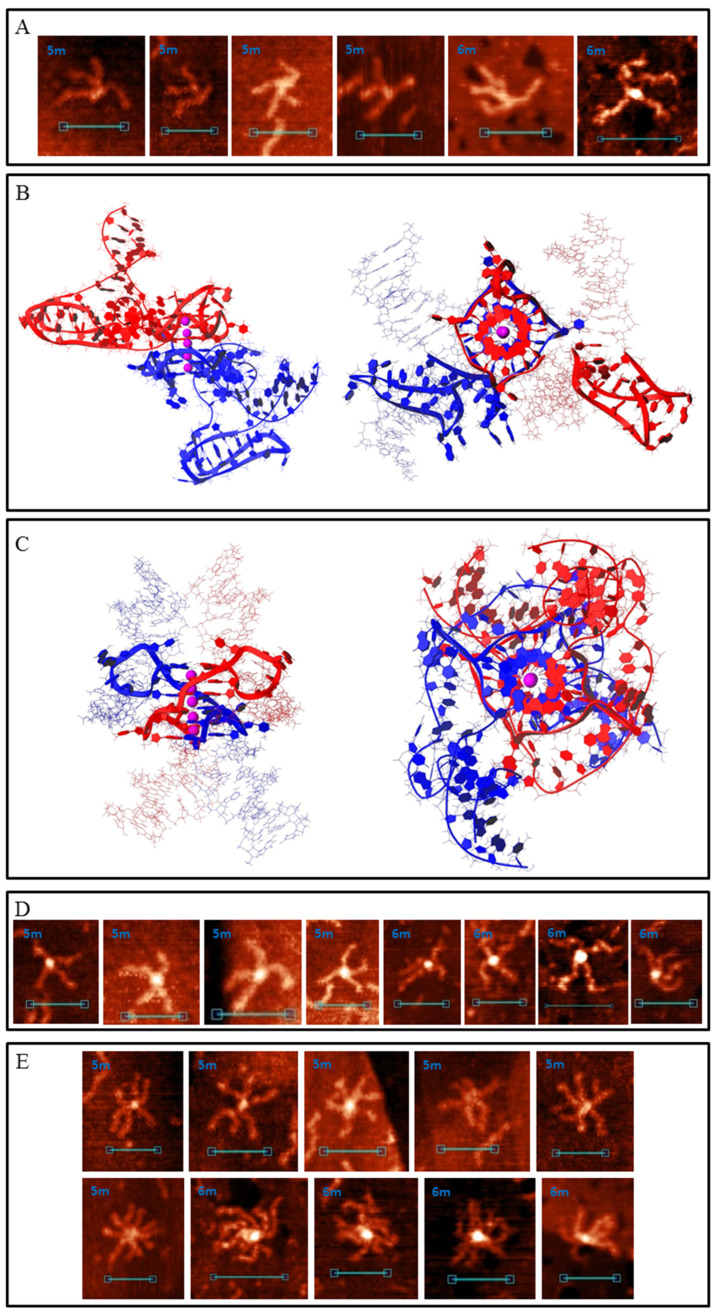
G4/IM-synaptic complexes formed by 5m and 6m samples. (**A**) Examples of bimolecular complexes, formed through stacking of intramolecular G4s which contain 5-base loops, revealed by AFM. Scale bars: 50 nm. (**B**) Molecular model of the structure, consistent with (**A**) (with two IMs folded). (**C**) Molecular model of duplex joining through the interlocked G4-dimer in which each G4 contains 5-base loops. (**D**) Examples of the complexes, consistent with model (**C**), revealed by AFM. Scale bars: 50 nm. (**E**) Examples of small multimeric G4/IM-synaptic complexes, formed by 5m and 6m samples.

Most common synaptic complex structures formed by the 5m and 6m samples were identical to those formed by the 4m sample (Figure 5 and Figure S3A). Complexes with structures depicted in Figure 3 were twice as rare. The AFM images of these complexes may differ from that of those formed by 2m, 3m and 4m samples only because different G_3_/C_3_-blocks may fold in these cases. Therefore, duplex arm lengths differ (particularly for complexes with structures of 2m and 3m forms, Figure S3B). The presence of an additional G_3_/C_3_-block may transform one or two (in the case of a 6m sample) middle blocks to a 5-base (TG_3_T/AC_3_A) or a 9-base loop. Such complexes based on intermolecular IMs (Figure 5C) are not distinguishable from the initial ones (Figure 5D) in AFM images, or they are absent, so we did not develop their molecular models. Complexes with one 5-base loop based on G4-G4 stacking (Figure 5E) in the AFM images were attributed to those shown in Figure 7A. Molecular modelling predicts that any inner G_3_-block may become a loop, but at the most stable variant (shown in Figure 7B) at one stacked G4 the second block (from the 5′-end) is looped out, and at the other G4 this is the fourth. Both IMs are folded in such a way that the third C_3_-blocks are looped out. These complexes are more stable than the initial ones shown in Figure 5. AFM also confirms that there are not so many pre-folded (or partly destroyed) examples, and one or both IMs are usually present. The quantity of folded IMs is distinguished by the core geometry (stretched in case of one IM and bent in case of two). Single-stranded not IM-folded loops are usually undetectable and only one example was found (right image in Figure 7A). The height of the core part was 1.2–1.4 nm. Molecular modelling indicates that for structures of this type with two long loops in each chain (6 m sample) at the G4–G4 core (as in the case of one 9-base loop), IM folding by central C3 blocks (side blocks serve as stems between IMs and other parts of the complex) without long loops is most energetically favourable (Figure S4). The AFM images of such complexes must be similar (may be not distinguishable) to that of those with one 5-base loop (Figure 7A). These may be a bit wider, but we did not find a wider 6 m sample structure with the same geometry, so it cannot be said exactly that they fold under the AFM conditions. Duplexes with at least one additional G_3_/C_3_-blocks may also join through an interlocked G4-dimer (Figure 5A), but the presence of 5-base loops and longer C-rich single chains that wrap G4-dimer (Figure 7C) widens the core in AFM images (2–2.5 nm height, Figure 7D). Synaptic complexes of such structures were abundant in the 5m and 6m samples. Analogically, the 6m sample is theoretically capable of forming structures with two 5-base loops at each G4 (or one 11-base loop), but AFM showed no synaptic complexes with the same geometry, but with a wider and higher (>2.5 nm) core part. It should be noted that there is also the possibility of folding several complexes with mixed topology, where, for example, one duplex forms a 5-base loop, but the second uses only neighbouring blocks in a synaptic complex formation. We did not model all these possibilities, partly due to their multiplicity, but instead we tried to consider the basic principles of their composition. For example, we may suggest that the large height variation of complexes depicted in Figure 7D (2–2.5 nm) is connected with the formation of such mixed structures.

Multimeric synaptic complexes, formed by 5m and particularly 6m samples, tended to grow and usually included dozens of duplexes. Even for relatively small complexes, including 3–5 molecules, each sample had its own structure that was formed by mixing different joining methods (Figure 7E).

In AFM images duplex arm lengths are generally equal, but not always. In some images, one or two arms are visibly shorter than the others. This is because they have bends or mainly melted tales.

**Figure 8:**
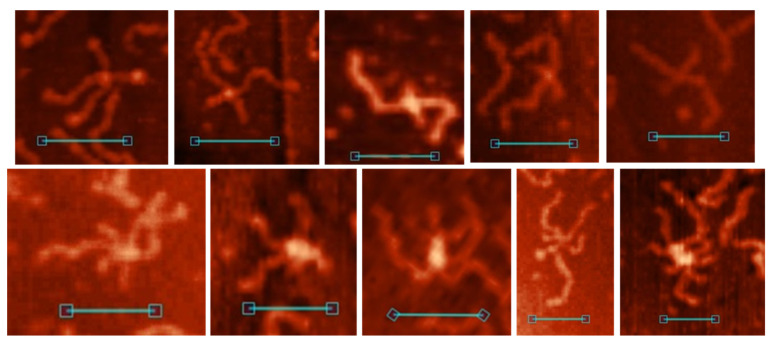
Di- and multimeric G4/IM-synaptic complexes, formed by 5s sample.

Moreover, 5s duplex (Table S1) was constructed using the same universal two-step PCR scheme (Figure 2). Five G_3_/C_3_-blocks in the PCR scheme were not located in the middle of the duplex molecule but shifted. AFM images indicate synaptic complexes formed by the 5 s sample (Figure 8). It is clearly visible that the joining crosshair also shifted, as for bimolecular and multimeric complexes. The duplex arm length ratio was about 4–7:1 (depending on which G_3_/C_3_-blocks participate in complex folding), which was consistent with the PQS position in the DNA sequence. This is an additional proof that duplexes are joined by G4/IM-synaptic complex folding.

In all molecular models, G4s have a parallel conformation, because this is a conformation of the (G_3_T)_3_G_3_ quadruplex at the single-strand level [53]. A molecular dynamic simulation was performed (50 ns) for all the molecular models presented here which proved their stability. Some typical energy landscapes of the molecular dynamic simulations are shown in Figure S5.

**Figure 9:**
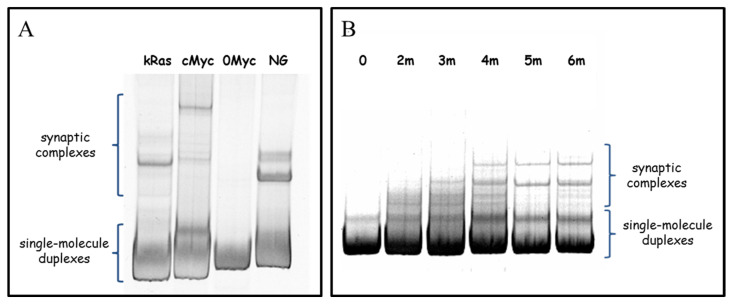
Non-denaturing PAGE of natural DNA amplicons (**A**) and model set of duplexes (**B**).

Formation of the synaptic complexes is an energy demanding process due to for the necessary unwinding of a GC-rich area. To additionally prove that this process occurs in solution, but not as a result of interaction with substrate surface, we checked the synaptic complexes formation by non-denaturing PAGE. The results are presented in Figure 9. All the natural duplex fragments (except 0Myc sample which is the only one that does not contain PQS) have additional low mobility PAGE bands (Figure 9A). These bands refer to G4/IM-synaptic complexes, spontaneously formed in solution. Analogously, a non-denaturing PAGE of the model set of duplexes (0–6m) revealed additional bands in all the samples, except 0 (Figure 9B). Diffuse PAGE bands, formed by 2m and 3m samples, indicate that the respective synaptic complexes are partly disrupted during PAGE. Sample 4 m has both clear and diffuse bands, suggesting the differing stability of different synaptic complexes. By non-denaturing PAGE it was previously shown that synaptic complexes may be formed by single-stranded G-rich DNA [33] and by synapsable duplexes that have non-complimentary “sticky” G-domains [34,35,36]. In this paper we show that natural complimentary duplexes can also spontaneously bind to one another through the formation of the G4/IM-synaptic complexes.

### 3.4. Antibody Analysis of the G4/IM-Synaptic Complexes

The above G4/IM-synaptic complex structures formed spontaneously from separate DNA duplexes, as determined by PCR. This method excluded Holliday structure (HS) formation, but one may note that, although our buffer did not contain Mg^2+^ ions, many of the synaptic structures were similar to HS in a stacked conformation [54] on AFM images. An antibody analysis was used to confirm the presence of G4 in the synaptic complexes.

**Figure 10:**
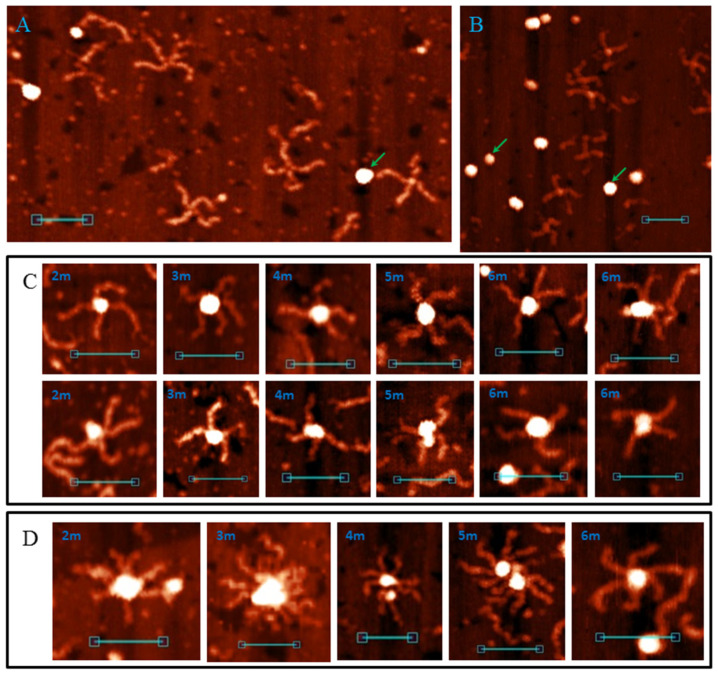
Interaction of the G4-specific antibody (1H6) with G4/IM-synaptic complexes and Holliday structure (HS). (**A**) AFM image of HS in extended conformation in the presence of 1H6 antibody; (**B**) AFM image of HS in a stacked form in the presence of 1H6 antibody; (**C**) Interaction of 1H6 antibody with cruciform G4/IM-synaptic complexes; (**D**) Interaction of 1H6 antibody with higher order synaptic complexes. Antibody molecules are marked with arrows.

We checked the affinity of the anti-G4 DNA antibody (clone 1H6; [24,25]) to model G4/IM-synaptic complex-forming sequences (2–6m) and synthesised immobile HS (see Materials and Methods and Figure S6). The 1H6 antibody (globes with height 3.5 ± 0.5 nm and diameter 8–17 nm) did not interact with the extended conformation (dominates in AFM buffer, Figure 10A) and the stacked forms (realised in presence of 10 mM Mg^2+^, Figure 10B) of the Holliday junction, but recognised most folded G4/IM-synaptic complex molecules for all 2–6m samples. The AFM images show the interaction of the antibody with the intersection points of the cruciforms (Figure 10C) and higher-order structures (Figure 10D). One or two antibody molecules interacted with the cruciforms, confirming the formation of a synaptic structure via G4–G4 stacking. This is observed for synaptic complexes formed by 4m, 5m, and 6m, but not by 2m and 3m. Not every cruciform was recognised by antibody molecules, supposedly due to folding via IMs and the absence of G4 motifs in these associates. We also observed HS formation at extended conformation (they are clearly distinguishable from synaptic complexes) in the AFM images, which was described in Section 3.5.

We also found that the G4-DNA antibody colocalised to the central regions of some duplexes, which did not participate in G4/IM-synaptic complex formation. This is similar to the confirmation of G4 folding in dsDNA, but statistically it happens no more often than the random colocalisation of antibody molecules at other parts of the DNA molecules. Therefore, we may conclude that the formation of the G4 structure in dsDNA is more likely when it folds as a part of the synaptic complex, rather than by itself.

### 3.5. G4/IM-Synaptic Complexes and Recombination

**Figure 11:**
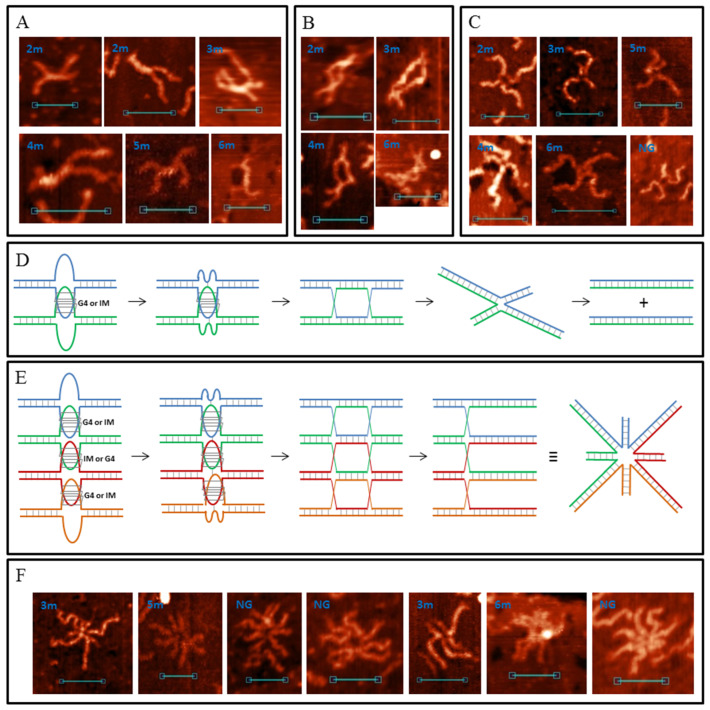
G4/IM-synaptic complex-promoted DNA strand exchange (recombination). (**A**) Examples of junctions with lengthy intersection points, revealed by AFM. (**B**) Structures with two HSs. (**C**) Junctions through one HS. Scale bars: 50 nm. (**D**) Schematic representation of the presumed mechanism of G4/IM-synaptic complex-promoted DNA strand exchange. (**E**) Presumed mechanism of tetra-HS formation from tetrameric G4/IM-synaptic complex. (**F**) Examples of multi-Holliday structures revealed by AFM. Scale bars: 50 nm.

As mentioned previously, G4/IM-synaptic complexes were not the only structures observed in the AFM images (Figure 11). Cruciform junctions with lengthy intersection points, “stretched bodies” (Figure 11A); molecular formations with two distinct HSs (Figure 11B); andtwo DNA molecules connected by just one HS (Figure 11C) were also observed. The height of “stretched bodies” is ~1.2 nm, and their length varies from 10 to 35 nm (shorter “stretched bodies” are not distinguishable from the IM-based synaptic complexes in the AFM images). HSs in Figure 11B were often stacked despite the lack of Mg^2+^ ions in AFM buffer. This may be due to the steric hindrance of the two extended HSs formed quite near each other. At junctions through one HS, they were always in an extended conformation. The formation of these structures was observed for every DNA duplex containing PQS (but not for the 0 and 0Myc samples). By analysing these images, we may suppose that they show different snapshots of one process, namely, the G4/IM-synaptic complex-mediated strand exchange (in vitro recombination). The suggested schematic mechanism for this process is shown in Figure 11D. The first step is the formation of base pairs (here AT pairs) between the G4 or IM loops and free complementary chains of the other duplex from the synaptic complex. Next, disrupting the synaptic complex structure leads not to the recovery of the initial two duplexes, but to confusion between the chains and the formation of two HSs. Their migration away from each other under in vitro conditions (in the absence of proteins mediating this process, such as RuvAB from *E. coli*) causes negative superhelicity of the DNA ring bordered by the HSs. Therefore, we may conclude that duplexes within HSs (Figure 11B) are partly unwound, contributing to the folding of noncanonical structures, for example, the emergence of Z-DNA areas or synaptic complexes (as in the 6 m sample, bottom image). Such folds with two distinct HSs are extremely rare and most molecules with negative superhelicity are supercoiled (Figure 11A) with different lengths and positions of the supercoiled part, which is consistent with spontaneous branch migration. Finally, when one HS reaches the end of the duplexes and resolves, superhelicity is lost and the duplexes are joined by one HS in an extended conformation (Figure 11C).

Analogously, we suggest that base pair formation between converged G4 or IM loops and complementary free chains from the other duplexes may also occur at multimeric synaptic complexes. Subsequent refolding of its structure and confusion between chains must lead to the formation of a multi-HS. This process for a four-duplex synaptic complex is schematically depicted in Figure 11E. We write about this multi-recombination process and multi-HS formation because such associates form even if they are extremely rare. All the AFM images are shown in Figure 11F. Most of them (three multi-HSs) were formed by NG sample which is a 200 bp amplicon of a G4-containing sequence located upstream of the *N. gonorrhoeae* pilin expression locus (pilE), and it is necessary for the initiation of nonreciprocal recombination between pilE and one of many silent pilin loci [7]. Based on our results, we may suggest that the process of pilin antigen switching goes through multimeric synaptic complex formation and its resolution to multi-HS. In the multi-Holliday structure (as well as in usual HS) in the extended conformation, long and short duplex arms must alternate (Figure 11E), but as clearly seen (particularly for the 3m sample) this does not always occur. Presumably, they are not fully extended but are partly stacked.

## 4. Discussion

We showed that DNA duplexes containing PQS may spontaneously form G4/IM-synaptic complexes. Such structures can be formed even by truncated PQS sequences. Therefore, there are more synaptic-forming sequences than predicted using different G4-finding software. Molecular modelling was used to elucidate the complex fine structures and the results confirmed the structures observed using AFM. G4 folding was confirmed through experiments with an anti-G4 DNA antibody. The mechanism of G4/IM-synaptic complex-mediated recombination was proposed.

The mechanism of synaptic complex formation should be further investigated. AFM experimental conditions (e.g., low ionic strength, particularly K^+^ concentration) do not contribute to G4 and IM folding. However, synaptic complex formation also reduces duplex stability which may contribute to synaptic complex folding. In vivo conditions (≈100 mM KCl, molecular crowding) stabilise duplexes and synaptic complexes [55,56,57]. Synaptic complex folding requires DNA duplex melting. The formation of right-handed crossovers may trigger such melting and lead to noncanonical secondary structure folding [58,59]. Most right-handed crosses require cytosine-phosphate group interactions at the anchoring point and are frequently stabilised by divalent cations (usually Mg^2+^). Our AFM buffer did not contain Mg^2+^ ions, so we propose that this lack is compensated by cytosine multiplicity at PQS sites but adding 10 mM MgCl_2_ to AFM buffer did not increase synaptic complex formation (Figure S7).

The stability of formed synaptic complexes depends on the PQS sequence, the sequence of its flanking regions [60] and environment (in vivo, it also includes protein factors, torsion stress and so on).

AFM spectroscopy showed that only about small percentages of duplexes containing PQS fold into G4/IM-synaptic complexes, but we also observed examples of varying degrees of synaptic complex decay (Figure S8). This means that their real quantity in the solution may be greater than that observed on the substrate. It is clearly visible that G/C-rich ssDNA chains are temporarily released during synaptic complex decomposition. Their formation may contribute to genome instability and double-strand breaks (DSB) formation.

In this study, we correlate AFM and molecular modelling data for only one simple G4/IM-pair (with single-base identical loops and only parallel G4 conformation). The diversity of synaptic complex structures is defined by the PQS sequence. For example, the diversity of possible synaptic complexes, formed by duplexes containing telomeric PQS, must increase because telomeric G4 is polymorphic (depending on the local environment, it may fold into parallel, antiparallel, or hybrid-type structures) [3]. The possibility of forming synaptic complexes by duplexes containing different PQSs (more probable cases in natural conditions) additionally diversifies possible structures.

One duplex pair may form diverse synaptic structures, and the tendency to form one or another type depends on the conditions, initial mutual arrangement, and the sequences. Different folded structures may bind to different protein factors, so synaptic complex heterogeneity may be a factor of uncertainty. It also serves as a fine-tuning element of biochemical processes.

## Figures and Tables

**Table 1:** Oligonucleotide sequences and MS data.

Code	Sequence	m/z [M+H]^+^, Found (Calculated)
R_NG	5′-TCATCTGCCGGTTGCATAGA	6108 (6108)
F_NG	5′-CAGTTTTAGTGCCGATTTTCGT	6722 (6722)
R_cMyc	5′-GTGGGCGGAGATTAGCGAGA	6288 (6287)
F_cMyc	5′-AATGGTAGGCGCGCGTAGTT	6213 (6213)
R_0Myc	5′-AAGTGGAGAGCTTGTGGACC	6223 (6222)
F_0Myc	5′-CACGGAAGTAATACTCCTCTCCTC	7232 (7232)
R_kRas	5′-CACCCTAGACCGCCCCAG	5374 (5374)
F_kRas	5′-CGCAGAGGGCAGAGCTATC	5862 (5862)
5′-fl	5′-GTAGTGAGACTCCTGAAAGAAGTATCTGACCAACTTACAGATAATATTAAAGCTCTACACGAGAC	20,043 (20,041)
3′-fl	5′-p-GTCATGTTCCTGACTTTAGACGTATCTGACCAACTTACAGATAAGGAATCCATCTAGACTCAGCG	20,023 (20,021)
mid_0	5′-p-CTCCAATGTGATCAGCTGCACGTATCTGACCAACTTACAGATAAGCCAAGTGTGATCAGCTGCAC	20,017 (20,016)
mid_2	5′-p-CTCCAATGTGATCAGCTGCACGTATCTGACCCACCCACAGATAAGCCAAGTGTGATCAGCTGCAC	19,962 (19,961)
mid_3	5′-p-CTCCAATGTGATCAGCTGCACGTATCTGACCCACCCACCCATAAGCCAAGTGTGATCAGCTGCAC	19,897 (19,897)
mid_4	5′-p-CTCCAATGTGATCAGCTGCACGTATCCCACCCACCCACCCATAAGCCAAGTGTGATCAGCTGCAC	19,843 (19,842)
mid_5	5′-p-CTCCAATGTGATCAGCTGCACGTCCCACCCACCCACCCACCCAAGCCAAGTGTGATCAGCTGCAC	19,788 (19,788)
mid_6	5′-p-CTCCAATGTGATCAGCTGCACCCCACCCACCCACCCACCCACCCGCCAAGTGTGATCAGCTGCAC	19,710 (19,709)
5′-fl_5	5′-p-GTAGTGAGACTCCTGAAAGAAGTCCCACCCACCCACCCACCCAATATTAAAGCTCTACACGAGAC	19,893 (19,892)
Lig1	5′-CTGATCACATTGGAGGTCTCGTGTAGAGCTT	9557 (9557)
Lig2	5′-AAGTCAGGAACATGACGTGCAGCTGATCAC	9250 (9249)
Pr	5′-GTAGTGAGACTCCTGAAAGAA	6503 (6503)
Pf	5′-CGCTGAGTCTAGATGGATTC	6149 (6148)
Hol-1	5′-p-TATCCATCGCTTGAATGGTCGACAATTGTTCCTTGTAATCTTTAGCGTTAATGACAGCAA	18,510 (18,508)
Hol-2	5′-p-GATTACAAGGAACAATTGTCCAACTCTTTATTTTCAGTGGTTTAGCGTTAATGACAGCAA	18,566 (18,565)
Hol-3	5′-p-CCACTGAAAATAAAGAGTTGAGGCGGTTGTTTTAGACAAATTTAGCGTTAATGACAGCAA	18,697 (18,697)
Hol-4	5′-p-TTTGTCTAAAACAACCGCCTGACCATTCAAGCGATGGATATTTAGCGTTAATGACAGCAA	18,530 (18,529)
Hol-fs	5′-p-CTGTAAGTTGGTCAGATACTTCTTTCAGGAGTCTCACTAC	12,332 (12,331)
Hol-fl	5′-GTAGTGAGACTCCTGAAAGAAGTATCTGACCAACTTACAGTTGCTGTCATTAACGCTAAA	18,490 (18,490)

## Supplementary Materials

The following supporting information can be downloaded at: https://www.mdpi.com/article/10.3390/polym14102118/s1, Figure S1: (A) Molecular model of tetrameric complex formed through intermolecular G4 and two IMs folding (not revealed by AFM). (B) Molecular model of trimolecular synaptic complex assembled through the formation of two intermolecular G4s. Theoretically, 3 m sample can form this structure, but it was not revealed by AFM.; Figure S2: Examples of di- and trimeric G4/IM-synaptic complexes formed by 4 m sample with the same structures as 2 m and 3 m form. Scale bars: 50 nm; Figure S3: Examples of simple cruciform synaptic complexes, formed by 5 m and 6 m samples. (A) Complexes with four G_3_T and/or C_3_A blocks involved. They have the same structures as the complexes formed by 4 m sample. (B) Complexes with only two G_3_T and/or C_3_A blocks involved. They have the same structures as formed by 2 m and 3 m samples. Scale bars: 50 nm; Figure S4: Molecular model of the possible complex, formed by 6 m sample through stacking of intramolecular G4s and containing two 5-base loops at each chain (not revealed by AFM); Figure S5: Evolution of the contributions to the free energy of the DNA. The plots were smoothed using the moving average method (span = 5). (A) In structure, shown in Figure 3A. (B) In structure, shown in Figure 3C. (C) In structure, shown in Figure 5A. (D) In structure, shown in Figure 5E. E_eq_—electrostatic energies, E_VDW_—Van der Waals energies, E_GB_—polar and E_surf_—non-polar contributions to the solvation energy, U—deformation energy of valence bonds, valence and dihedral angles; Figure S6: Scheme of immobile Holliday structure synthesis; Figure S7: Examples of broken G4/IM-synaptic complexes; Figure S8: Examples of broken G4/IM-synaptic complexes; Table S1: Sequences of natural and model DNA-duplexes used in AFM experiments. Yellow highlighted are PQSs and their complimentary C-rich chains.
