# Supplementary material for: Spontaneous DNA Synapsis by Forming Noncanonical Intermolecular Structures
Source: Polymers (Basel). 2022 May 23;14(10):2118. doi: 10.3390/polym14102118 (PMC9144187; doi:10.3390/polym14102118)
Supplement: Supplementary file 1 [file polymers-14-02118-s001.zip › polymers-1710406-supplementary.pdf]

Article

Spontaneous DNA Synapsis by Forming Noncanonical Intermolecular Structures

Viacheslav Severov [1,*,†], Vladimir Tsvetkov [1,2,3,*,†], Nikolay Barinov [1], Vladislav Babenko [1], Dmitry Klinov [1,4] and Galina Pozmogova [1]

[1] Federal Research and Clinical Center of Physical-Chemical Medicine, Malaya Pirogovskaya Str. 1a, Moscow 119435, Russia; nik_mipt@mail.ru (N.B.); daniorerio34@gmail.com (V.B.); klinov.dmitry@mail.ru (D.K.); pozmge@gmail.com (G.P.)

[2] Institute of Biodesign and Complex System Modeling, I.M. Sechenov First Moscow State Medical University, Trubetskaya Str. 8-2, Moscow 119991, Russia

[3] A.V. Topchiev Institute of Petrochemical Synthesis, Leninsky prospect Str. 29, Moscow 119991, Russia

[4] Peoples' Friendship University of Russia (RUDN University), 6 Miklukho-Maklaya str.6, 117198 Russian Federation

* Correspondence: wws83@yandex.ru (V.S.); v.b.tsvetkov@gmail.com (V.T.)

† These authors contributed equally to this work.

Table S1. Sequences of natural and model DNA-duplexes used in AFM experiments. Yellow highlighted are PQSs and their complimentary C-rich chains.

cMyc	5′AATGGTAGGCGCGCGTAGTTAATTCATGCGGCTCTCTTACTCTGTTTACATCCTAGAGCTAGAGTGCTCGGCTGCCCGGCTGAGTCTCCTCCCCAC<br>CTTCCCCACCCTCCCCACCCTCCCCATAAGCGCCCCTCCCGGGTTCCCAAAGCAGAGGGCGTGGGGGAAAAGAAAAAAGATCCTCTCTCGCTAATC<br>TCCGCCCAC<br>3′TTACCATCCGCGCGCATCAATTAAGTACGCCGAGAGAATGAGACAAATGTAGGATCTCGATCTCACGAGCCGACGGGCCGACTCAGAGGAGGG<br>GTGGAAGGGGTGGGAGGGGTGGGAGGGGTATTCGCGGGGAGGGCCCAAGGGTTTCGTCTCCCGCACCCCCTTTTCTTTTTTCTAGGAGAGAGCGAT<br>TAGAGGCGGGTG
kRas	5′CGCAGAGGGCAGAGCTATCGATGCGTTCCGCGCTCGATTCTTCTTCAGACGGGCGTACGAGAGGGAGCGGCTGAGGGCGGTGTGGGAAGAGGGA<br>AGAGGGGGAGGCAGCGAGCGCCGGCGGGGAGAAGGAGGGGGCCGGGCCGGGCCGGCGGGGGAGGAGCGGGGGCCGGGCCGGCGGAGGAAGG<br>GGTGGCTGGGGCGGTCTAGGGTG<br>3′GCGTCTCCCGTCTCGATAGCTACGCAAGGCGCGAGCTAAGAAGAAGTCTGCCCGCATGCTCTCCCTCGCCGACTCCCGCCACACCCTTCTCCCTTC<br>TCCCCCTCCGTCGCTCGCGGCCGCCCCTCTTCCTCCCCCGGCCCGGCCCGGCCGCCCCCTCCTCGCCCCCGGCCCGGCCGCCTCCTTCCCCACCGAC<br>CCCGCCAGATCCCAC
NG	5′CAGTTTTAGTGCCGATTTTCGTTACTTTTTTATTGGCATGGGGTATCGGGTGTGTTGATTGGGTCGGAATTTGAGATTTTTGAATTTACGCGTTAGAA<br>TAGGGTGGGTTGGGTGGGGAATTTTCTATTTTTTAAAAAGCTCCGTTTTCTTGGAAAGCATTTGAAATCGGCGCGTGGTGTTTCTATGCAACCGGCAG<br>ATGA<br>3′GTCAAAATCACGGCTAAAAGCAATGAAAAAATAACCGTACCCCATAGCCCACACAACTAACCCAGCCTTAAACTCTAAAAACTTAAATGCGCA<br>ATCTTATCCCACCCAACCCACCCCTTAAAAGATAAAAAATTTTTCGAGGCAAAAGAACCTTTCGTAAACTTTAGCCGCGCACCACAAAGATACGTT<br>GGCCGTCTACT
0Myc	5′CACGGAAGTAATACTCCTCTCCTCTTCTTTGATCAGAATCGATGCATTTTTTGTGCATGACCGCATTTCCAATAATAAAAGGGGAAAGAGGACCTG<br>GAAAGGAATTAAACGTCCGGTTTGTCCGGGGAGGAAAGAGTTAACGGTTTTTTTCACAAGGGTCTCTGCTGACTCCCCCGGCTCGGTCCACAAGCT<br>CTCCACTT<br>3′GTGCCTTCATTATGAGGAGAGGAGAAGAAACTAGTCTTAGCTACGTAAAAAACACGTACTGGCGTAAAGGTTATTATTTTCCCCTTTCTCCTGGAC<br>CTTTCCTTAATTTGCAGGCCAAACAGGCCCCTCCTTTCTCAATTGCCAAAAAAAGTGTTCCCAGAGACGACTGAGGGGGCCGAGCCAGGTGTTCGA<br>GAGGTGAA
0	5′GTAGTGAGACTCCTGAAAGAAGTATCTGACCAACTTACAGATAATATTAAAGCTCTACACGAGACCTCCAATGTGATCAGCTGCACGTATCTGAC<br>CAACTTACAGATAAGCCAAGTGTGATCAGCTGCACGTCATGTTCCTGACTTTAGAC-<br>GTATCTGACCAACTTACAGATAAGGAATCCATCTAGACTCAGCG<br>3′CATCACTCTGAGGACTTTCTTCATAGACTGGTTGAATGTCTATTATAATTTCGAGATGTGCTCTGGAGGTTACACTAGTCGACGTGCATAGACTGGT<br>TGAATGTCTATTCGGTTCACACTAGTCGACGTGCAGTACAAGGACTGAAATCTGCATAGACTGGTTGAATGTCTATTCCTTAGGTAGATCTGAGTCG<br>C

2 m	5′GTAGTGAGACTCCTGAAAGAAGTATCTGACCAACTTACAGATAATATTAAAGCTCTACACGAGACCTCCAATGTGATCAGCTGCACGTATCTGAC<br>CCACCCACAGATAAGCCAAGTGTGATCAGCTGCACGTCATGTTCCTGACTTTAGAC-<br>GTATCTGACCAACTTACAGATAAGGAATCCATCTAGACTCAGCG<br>3′CATCACTCTGAGGACTTTCTTCATAGACTGGTTGAATGTCTATTATAATTTCGAGATGTGCTCTGGAGGTTACACTAGTCGACGTGCATAGACTGG<br>GTGGGTGTCTATTCGGTTCACACTAGTCGACGTGCAGTACAAGGACTGAAATCTGCATAGACTGGTTGAATGTCTATTCCTTAGGTAGATCTGAGTC<br>GC
3 m	5′GTAGTGAGACTCCTGAAAGAAGTATCTGACCAACTTACAGATAATATTAAAGCTCTACACGAGACCTCCAATGTGATCAGCTGCACGTATCTGAC<br>CCACCCACCCATAAGCCAAGTGTGATCAGCTGCACGTCATGTTCCTGACTTTAGAC-<br>GTATCTGACCAACTTACAGATAAGGAATCCATCTAGACTCAGCG<br>3′CATCACTCTGAGGACTTTCTTCATAGACTGGTTGAATGTCTATTATAATTTCGAGATGTGCTCTGGAGGTTACACTAGTCGACGTGCATAGACTGG<br>GTGGGTGGGTATTCGGTTCACACTAGTCGACGTGCAGTACAAGGACTGAAATCTGCATAGACTGGTTGAATGTCTATTCCTTAGGTAGATCTGAGTC<br>GC
4 m	5′GTAGTGAGACTCCTGAAAGAAGTATCTGACCAACTTACAGATAATATTAAAGCTCTACACGAGACCTCCAATGTGATCAGCTGCACGTATCCCAC<br>CCACCCACCCATAAGCCAAGTGTGATCAGCTGCACGTCATGTTCCTGACTTTAGAC-<br>GTATCTGACCAACTTACAGATAAGGAATCCATCTAGACTCAGCG<br>3′CATCACTCTGAGGACTTTCTTCATAGACTGGTTGAATGTCTATTATAATTTCGAGATGTGCTCTGGAGGTTACACTAGTCGACGTGCATAGGGTGG<br>GTGGGTGGGTATTCGGTTCACACTAGTCGACGTGCAGTACAAGGACTGAAATCTGCATAGACTGGTTGAATGTCTATTCCTTAGGTAGATCTGAGTC<br>GC
5 m	5′GTAGTGAGACTCCTGAAAGAAGTATCTGACCAACTTACAGATAATATTAAAGCTCTACACGAGACCTCCAATGTGATCAGCTGCACGTCCCACCC<br>ACCCACCCACCCAAGCCAAGTGTGATCAGCTGCACGTCATGTTCCTGACTTTAGAC-<br>GTATCTGACCAACTTACAGATAAGGAATCCATCTAGACTCAGCG<br>3′CATCACTCTGAGGACTTTCTTCATAGACTGGTTGAATGTCTATTATAATTTCGAGATGTGCTCTGGAGGTTACACTAGTCGACGTGCAGGGTGGGT<br>GGGTGGGTGGGTTCGGTTCACACTAGTCGACGTGCAGTACAAGGACTGAAATCTGCATAGACTGGTTGAATGTCTATTCCTTAGGTAGATCTGAGTC<br>GC
6 m	5′GTAGTGAGACTCCTGAAAGAAGTATCTGACCAACTTACAGATAATATTAAAGCTCTACACGAGACCTCCAATGTGATCAGCTGCACCCCACCCAC<br>CCACCCACCCACCCGCCAAGTGTGATCAGCTGCACGTCATGTTCCTGACTTTAGAC-<br>GTATCTGACCAACTTACAGATAAGGAATCCATCTAGACTCAGCG<br>3′CATCACTCTGAGGACTTTCTTCATAGACTGGTTGAATGTCTATTATAATTTCGAGATGTGCTCTGGAGGTTACACTAGTCGACGTGGGGTGGGTGG<br>GTGGGTGGGTGGGCGGTTCACACTAGTCGACGTGCAGTACAAGGACTGAAATCTGCATAGACTGGTTGAATGTCTATTCCTTAGGTAGATCTGAGT<br>CGC
5 s	5′GTAGTGAGACTCCTGAAAGAAGTCCCACCCACCCACCCACCCAATATTAAAGCTCTACACGAGACCTCCAATGTGATCAGCTGCACGTATCTGAC<br>CAACTTACAGATAAGCCAAGTGTGATCAGCTGCACGTCATGTTCCTGACTTTAGAC-<br>GTATCTGACCAACTTACAGATAAGGAATCCATCTAGACTCAGCG<br>3′CATCACTCTGAGGACTTTCTTCAGGGTGGGTGGGTGGGTGGGTTATAATTTCGAGATGTGCTCTGGAGGTTACACTAGTCGACGTGCATAGACTGG<br>TTGAATGTCTATTCGGTTCACACTAGTCGACGTGCAGTACAAGGACTGAAATCTGCATAGACTGGTTGAATGTCTATTCCTTAGGTAGATCTGAGTC<br>GC

polymers

MDPI

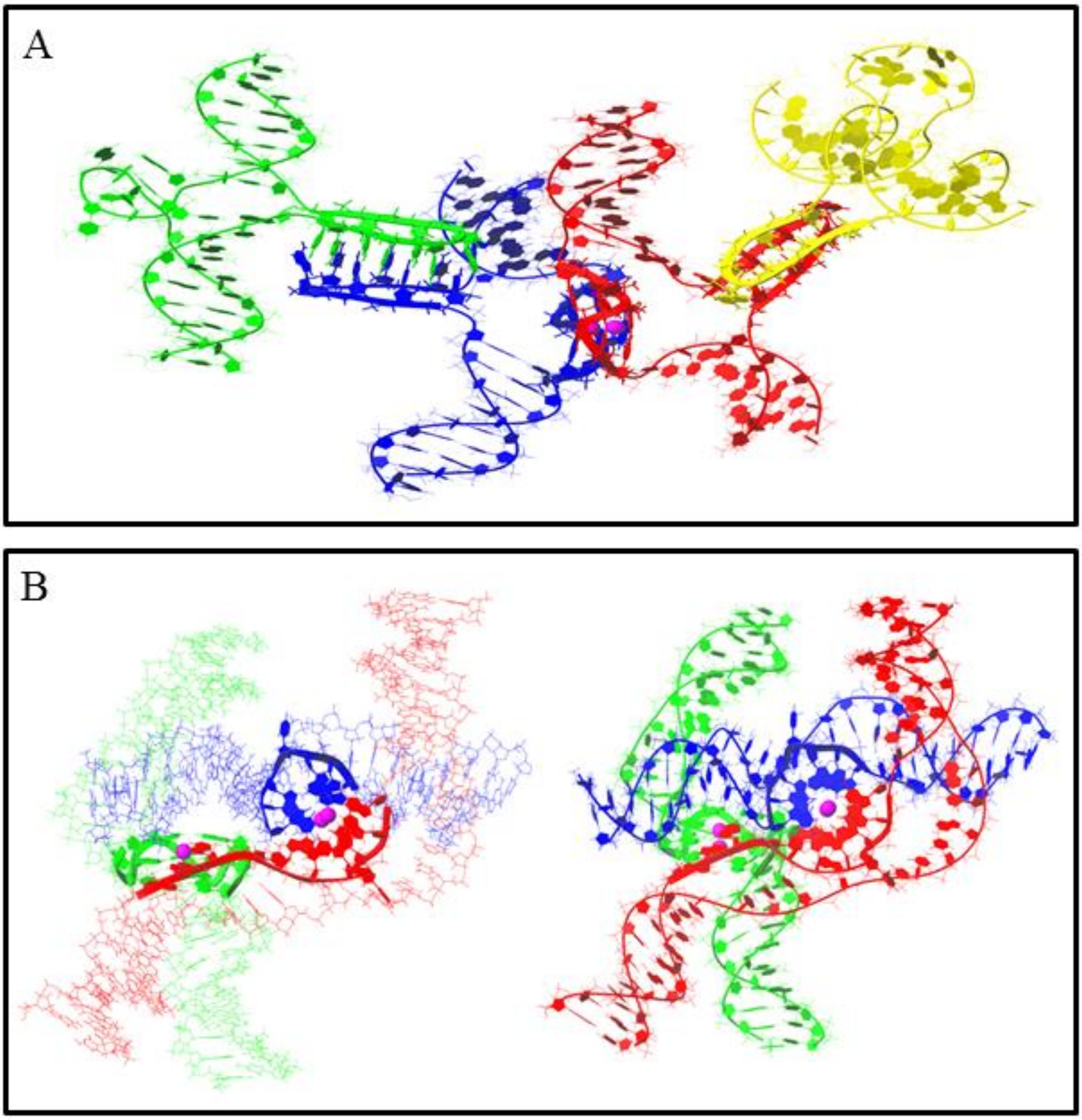

Figure S1. Examples of di- and trimeric G4/IM-synaptic complexes, formed by 4m sample, with the same structures as 2m and 3m form. Scale bars: 50 nm.

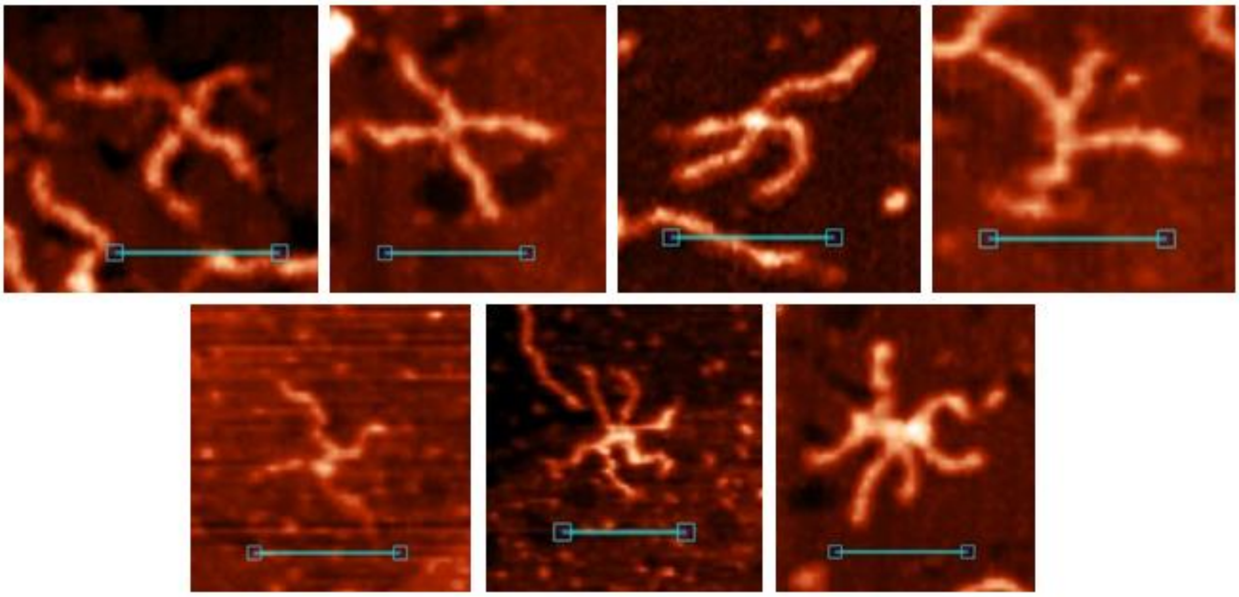

Figure S2. Examples of di- and trimeric G4/IM-synaptic complexes, formed by 4m sample, with the same structures as 2m and 3m form. Scale bars: 50 nm.

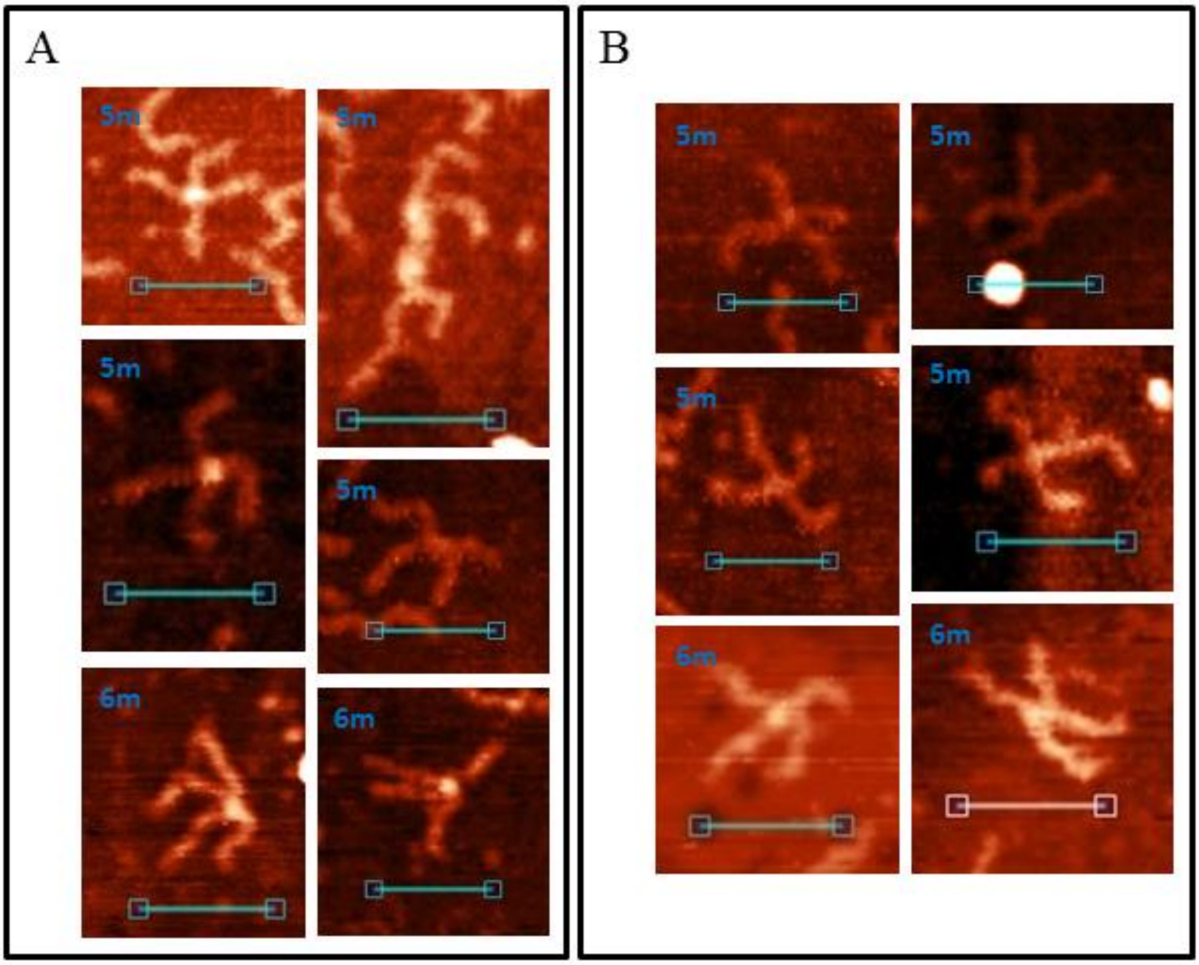

Figure S3. Examples of simple cruciform synaptic complexes, formed by 5m and 6m samples. (**A**) Complexes with four G_3T and/or C_3A blocks involved. They have the same structures as the complexes formed by 4m sample. (**B**) Complexes with only two G_3T and/or C_3A blocks involved. They have the same structures as formed by 2m and 3m samples. Scale bars: 50 nm.

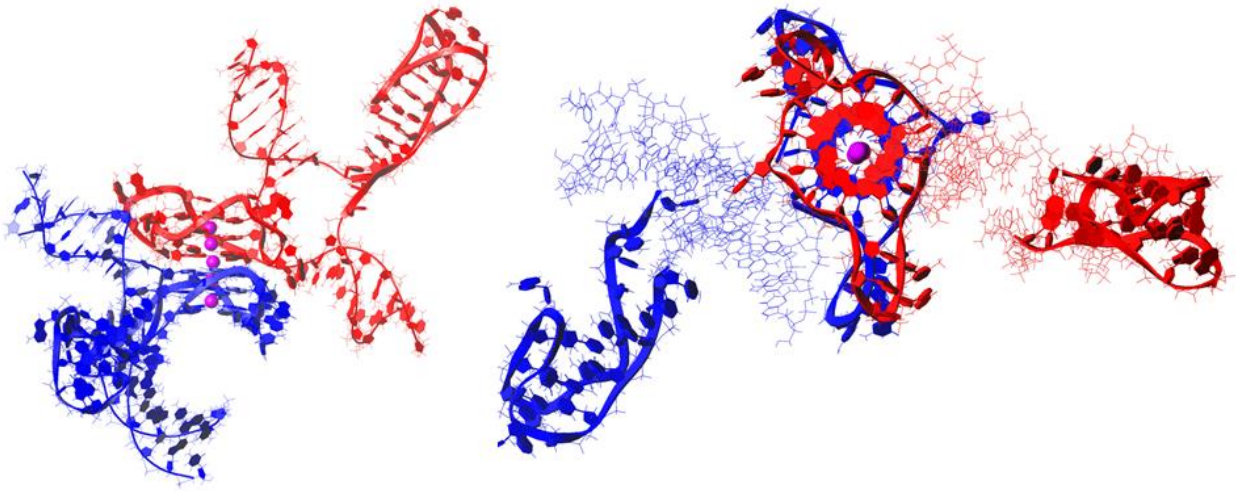

Figure S4. Molecular model of the possible complex, formed by 6m sample through stacking of intramolecular G4s and containing two 5-base loops at each chain (not revealed by AFM).

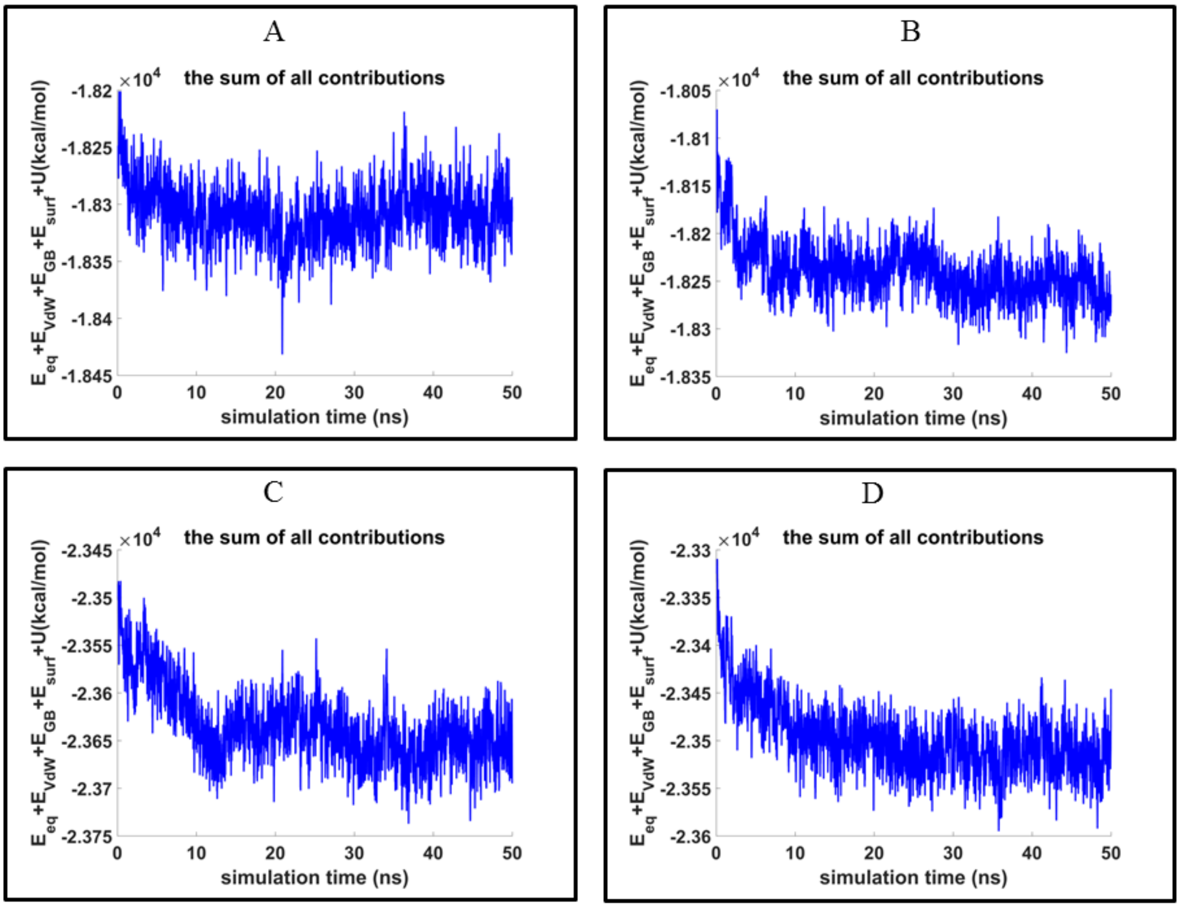

Figure S5. Evolution of the contributions to the free energy of the DNA. The plots were smoothed using the moving average method (span = 5). (**A**) In structure, shown in Fig. 3A. (**B**) In structure, shown in Fig. 3C. (**C**) In structure, shown in Fig. 5A. (**D**) In structure, shown in Fig. 5E. E_{eq} – electrostatic energies, E_{VDW} – Van der Waals energies, E_{GB} – polar and E_{surf} – non-polar contributions to the solvation energy, U – deformation energy of valence bonds, valence and dihedral angles.

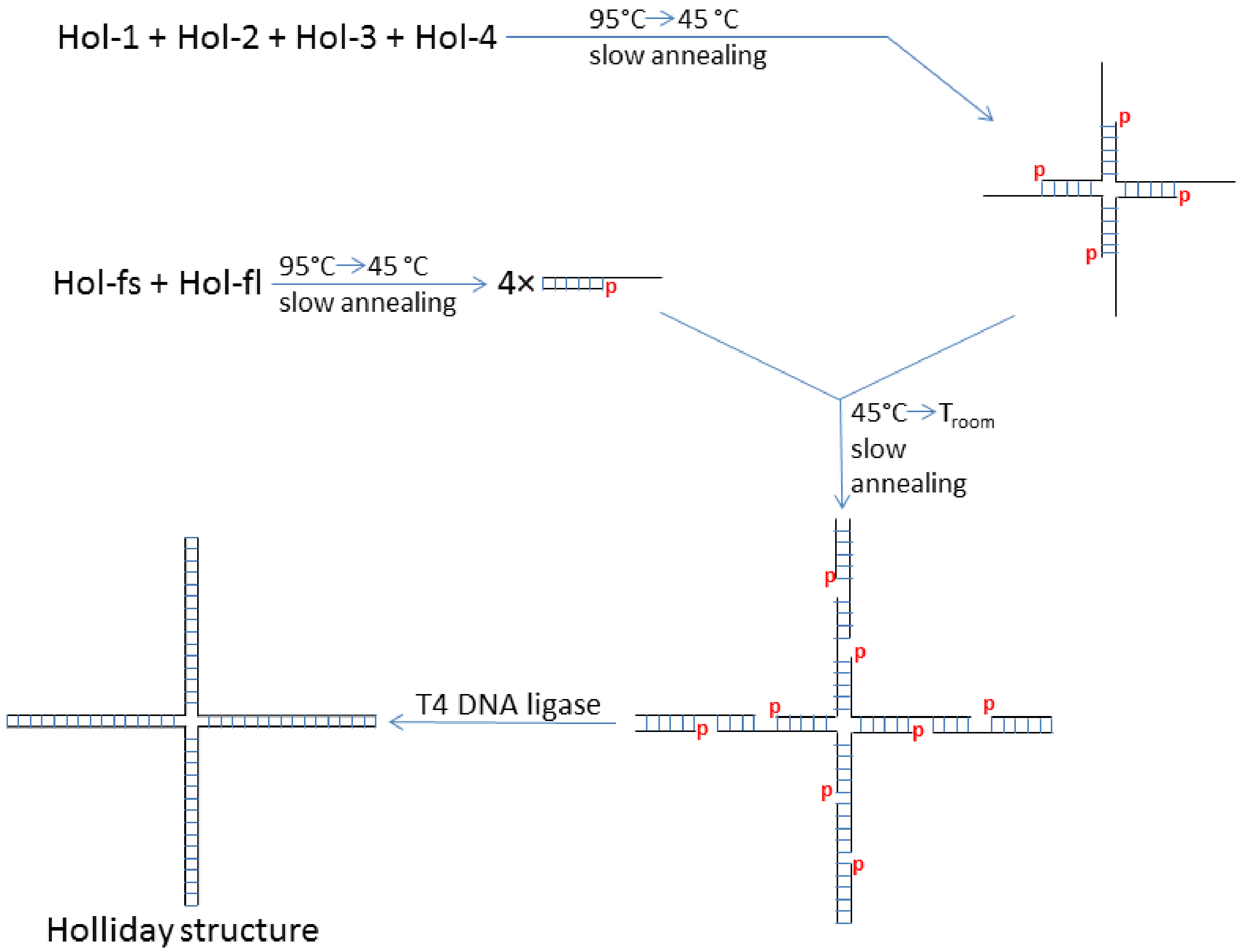

Figure S6. Scheme of immobile Holliday structure synthesis.

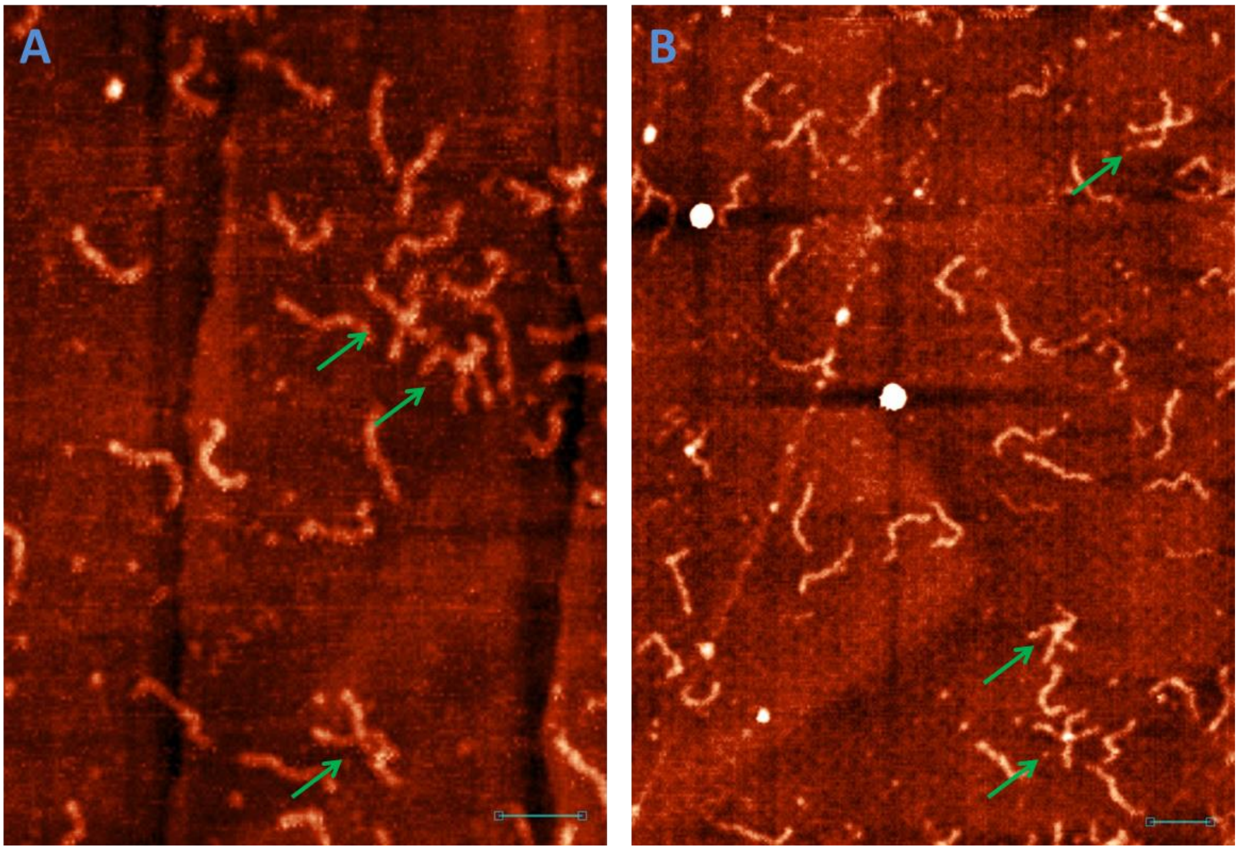

Figure S7. AFM images of 5m sample under different conditions. (**A**) AFM-buffer (10 mM KCl, 10 mM Tris-HCl, pH 5.6. (**B**) AFM-buffer with addition of 10 mM $MgCl_2$. The green arrows show the folded G4/IM-synaptic complexes. Scale bars: 50 nm.

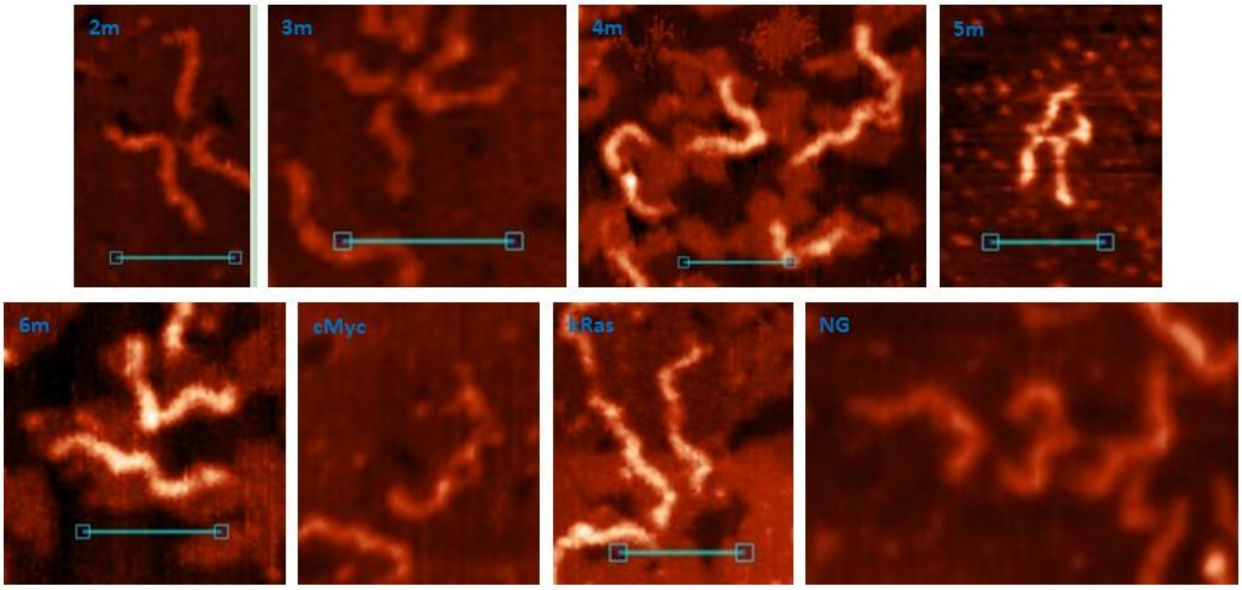

Figure S8. Examples of broken G4/IM-synaptic complexes.
